# Indivisibilities in investment and the role of a capacity market

**DOI:** 10.1007/s11149-024-09473-6

**Published:** 2024-03-07

**Authors:** Nicolas Stevens, Yves Smeers, Anthony Papavasiliou

**Affiliations:** 1 Center for Operations Research and Econometrics, UCLouvain, Louvain-la-Neuve, Belgium; 2 School of Electrical and Computer Engineering, National Technical University of Athens, Athens, Greece

**Keywords:** Pricing indivisibilities, Investment problem, Capacity market, Convex hull pricing, C61, D41, D44, D47, D50, L51, Q41

## Abstract

The topic of pricing non-convexities in power markets has been explored vividly in the literature and among practitioners for the past twenty years. The debate has been focused on indivisibilities in short-term auctions, the computational tractability of some pricing proposals, and the economic analysis of their behavior. In this paper, we analyse a source of non-convexities that is not discussed as broadly: the indivisibilities in investment decisions. The absence of equilibrium that we are primarily concerned about is the *long-term* equilibrium. We derive a capacity expansion model with indivisibilities and we highlight the issues arising from it. We discuss its relevance and address one particular argument for neglecting indivisibilities in investment, namely market size. We investigate to what extent a capacity market that clears discrete offers can mitigate the lumpiness problem. We particularly introduce the novel concept of convex hull pricing for capacity auctions. We illustrate the main findings with a numerical experiment conducted on the capacity expansion model used by ENTSO-E to assess the adequacy of the entire European system.

**Supplementary Information:**

The online version contains supplementary material available at 10.1007/s11149-024-09473-6.

## Introduction

The restructuring of the electricity industry is work in progress for more than 25 years. Early discussions mainly concentrated on decentralized versus centralized organizations of the market (Stoft, 2002). The latter system emerged, and with it the idea of a centralized market clearing, and a dispatch associated to the so-called merit order of plants that reflects fuel costs. It was quickly recognized that generation plants are also characterized by “indivisibilities”, such as start-up cost or minimum time between shutdown and startup. Accounting for these aspects required replacing the merit order-based dispatch by a unit commitment. This invalidated the clean neoclassical interpretation of electricity prices that, according to the doxa, supports competition in the industry. This made the market design more complex and generated a lot of implementation and methodological work, including a vivid debate in the literature and among practitioners about the right way to price in power auctions (O’Neill, Sotkiewicz, Hobbs, Rothkopf, and Stewart Jr, 2005; Hogan & Ring, 2003).

Indivisibilities also have a long-term dimension. In the same way that plants go through short-run cycles where they are started, operated for some hours, and shut down, they also go through a long-term cycle where they are built, operated over several years, and are eventually dismantled. Each of these stages implies costs that, once incurred, become stranded, hence constituting indivisibilities. In contrast with the short-run market, long-run indivisibilities did not receive much attention, whether in the literature or in practice, so far. A notable exception is Scarf’s ground-breaking paper (Scarf, 1994), which recognizes the indivisibilities in the choice of technologies as an issue.

The apparent neglect of indivisibilities in long-term electricity markets contrasts with the attention given to market failures and how these interact with investment incentives. The notion of “missing money” has been central in these discussions since Joskow (2007) enlightened the debate on the subject. The author relies on a stylized capacity expansion model to show that long-term elements are missing in the short-run market, which makes it unable to send adequate investment signals. The missing money debate generated considerable but often inconclusive discussions on the respective merits of different market designs such as energy-only markets and capacity markets. The energy transition in Europe and its implication of fully restructuring the capital stock of the generation system gave a new impetus to the subject. It was recognized that the insufficient incentive to invest was rooted not only in “missing money” but also in missing and incomplete financial markets, which are more difficult issues to explore and remedy. Considerable work has been undertaken in the UK since at least 2013 (UK Department of Energy & Climate Change, 2013; Grubb & Newbery, 2018; Helm, 2017). This led to the idea of using instruments such as contracts for differences (CfDs), power purchase agreements (PPAs) and capacity auctions (CRM) to mitigate this missing incentive for investment (De Maere d’Aertrycke, Ehrenmann, and Smeers, 2017). The general principle of this approach is that existing markets should be complemented by additional market instruments targeted at the incentive to invest. The war in Ukraine and the new European policy of moving away from Russian gas supplies reinforced the sense of urgency of the investment problem. This led to an explosion of papers to remedy not only the impact of high gas prices on the power market but also the possibly insufficient incentives to invest. It reinforced the push for the already mentioned market instruments (CfDs, PPAs, CRM).

Our paper aims at contributing to this literature on investment incentives, but focusing on the—much less discussed—problem of non-convexities in investment. Some papers have focused on the effect of the *short-run* non-convexities on the investment incentives (Mays, Morton, and O’Neill, 2021; Byers & Hug, 2023). Instead, our work focuses on the non-convexities in the investment itself. The goal of an investor to maximize profits still remains the same in the presence of indivisibilities, but these indivisibilities can distort the capacity mix that results from existing incentives. As in Joskow’s initial analysis of missing money (Joskow, 2007), we examine the problem through a deterministic capacity expansion model. This corresponds to a complete market (no uncertainty or missing market) and thus makes it possible to focus on the sole effect of indivisibilities. We analyse the possibility that, very much like indivisibilities in the short-run market required a regulatory authority to clear the short-term market, long-term indivisibilities may also require such an authority to coordinate investment. Our formal analysis leads to ideas related to the work of French economists Finon and Roques that claim that there exist fundamental difficulties for coordinating investment in the restructured power market, with the conclusion that direct regulated public intervention should be introduced for that purpose (Roques & Finon, 2017; Finon & Beeker, 2022). The authors refer to this mix of market and public coordination as the “hybrid market”—a notion also supported by Joskow (2022).

In Sects. 2 and 3, we introduce a long-term investment model, and we analyse the effects of the market imperfection at work, namely indivisibilities or non-convexities in investment decisions. We show that indivisibilities in investment result in a distortion of incentives for the individual market agents—a long-term *lost opportunity cost*, similarly to what happens in a short-term market with indivisibilities. Is this *long-term* lost opportunity cost important? In Sect. 4, we derive one theoretical argument, inspired from the theory of general equilibrium, for neglecting indivisibilities in general, and then discuss its relevance to the investment problem. We show that the issue stemming from discrete investment, under certain pricing approaches, may be arbitrarily large. In principle, the discussion of the first part of the paper (Sects. 2, 3 and 4) applies to any industry. In practice, however, one may expect the problem to be more severe in the electricity sector. Because there are important technical barriers to the storage and transportation of electricity, a shortfall of generation capacity in a given location may not be compensated either by a stock of energy or by raising imports. Unlike many industries, electricity has hardly any means to react to a local shortage of production capacity. These supply and transportation rigidities, combined with an electricity demand which has to be met just in time by production, and which is notably inelastic, especially in the short run, may exacerbate the impact of investment indivisibilities on energy prices—therefore on investment incentives.

The second part of the paper focuses on the interplay between capacity markets and investment indivisibilities. In Sect. 5, we analyse to what extent the long-term lost opportunity cost can be corrected by market mechanisms. Capacity markets are one way to coordinate long-term investments. Some existing capacity markets acknowledge, in their design, the indivisible nature of investment decisions. For example, the Belgian capacity market *only* includes indivisible bids [Elia (2022), art. 235, sec. 6.2]. But if the benefits of a capacity market as an instrument to hedge investment risk (De Maere d’Aertrycke, Ehrenmann, and Smeers, 2017) or to mitigate market power in the energy market (Fabra, 2018) have been well analysed [see also (Stoft, 2002; Cramton & Stoft, 2005; Cramton, Ockenfels, and Stoft, 2013)], little has been said about the effect of the capacity market on the incentives of the agents to invest in a market with long-term indivisibilities. Indeed, if lumpiness of investment has sporadically been mentioned to justify CRMs (Mastropietro, Rodilla, and Batlle, 2017), no formal discussion of the argument has been provided so far to the best of our knowledge. We analyse to what extent a capacity market could turn out to be a tool that mitigates the *long-term* lost opportunity costs stemming from indivisibilities, or if it alternatively exacerbates them. We particularly discuss the design of a CRM under discrete offers and we introduce the concept of *convex hull pricing* for capacity auctions. Finally, Sect. 6 illustrates our findings with a model of the European system. We perform our simulations with the European Resource Adequacy Assessment (ERAA) model used by ENTSO-E (2021) to estimate the need for investments in Europe.

## The continuous investment problem

The *continuous* investment problem provides us with a useful benchmark. Its analysis was pioneered by Boiteux (1960), who showed that *marginal pricing* provides market agents with the right incentives to invest in the welfare-maximizing generation mix. The analysis resolves the fallacy according to which a peaking unit could not possibly cover its fixed cost by solely relying on market payments. The analysis of Boiteux can be illustrated by considering the following long-term *continuous* investment model (which admits a decentralized interpretation):

1a $$\begin{aligned} \max _{q,x,d \ge 0} ~~&\sum _{t \in {\mathcal {T}}} \Delta T_t V_t d_t - \sum _{g \in {\mathcal {G}}} \sum _{t \in {\mathcal {T}}} \Delta T_t MC_g q_{g,t} - \sum _{g \in {\mathcal {G}}} IC_g x_g \end{aligned}$$

1b $$\begin{aligned} (\Delta T_t \pi _t) ~~&d_t \le \sum _{g \in {\mathcal {G}}} q_{g,t} ~~~ \forall t \in {\mathcal {T}} \end{aligned}$$

1c $$\begin{aligned} (\Delta T_t \mu _{g,t}) ~~&q_{g,t} \le x_g ~~~ \forall g \in {\mathcal {G}}, ~ t \in {\mathcal {T}} \end{aligned}$$

1d $$\begin{aligned} (\Delta T_t \eta _t) ~~&d_t \le D_t ~~~ \forall t \in {\mathcal {T}} \end{aligned}$$

The variables $x_g$, $q_{g,t}$ and $d_t$ stand respectively for the investment in technology $g \in {\mathcal {G}}$, the actual production from technology *g* at period $t \in {\mathcal {T}}$, and the consumption of energy at period *t*. Investment cost is indicated as $IC_g$, while marginal cost is indicated as $MC_g$. The total served demand at period *t*, $d_t$, is valued at $V_t$, which is assumed to be the *right* value of lost load (VOLL), cf. Stoft (2002). $D_t$ is the observed load while $\Delta T_t$ stands for the duration of period *t*. As indicated by the inequality in the market clearing constraint (1b), we assume *free disposal*. The optimality conditions of problem (1) are:

2a $$\begin{aligned} 0 \le q_{g,t} ~&\perp ~ MC_g - \pi _t + \mu _{g,t} \ge 0&\forall g \in {\mathcal {G}}, ~ t \in {\mathcal {T}} \end{aligned}$$

2b $$\begin{aligned} 0 \le x_g ~&\perp ~ IC_g - \sum _{t \in {\mathcal {T}}} \Delta T_t \mu _{g,t} \ge 0&\forall g \in {\mathcal {G}} \end{aligned}$$

2c $$\begin{aligned} 0 \le d_t ~&\perp ~ -V_t + \pi _t + \eta _t \ge 0&\forall t \in {\mathcal {T}} \end{aligned}$$

2d $$\begin{aligned} 0 \le x_g - q_{g,t} ~&\perp ~ \mu _{g,t} \ge 0&\forall g \in {\mathcal {G}}, ~ t \in {\mathcal {T}} \end{aligned}$$

2e $$\begin{aligned} 0 \le D_t - d_t ~&\perp ~ \eta _t \ge 0&\forall t \in {\mathcal {T}} \end{aligned}$$

2f $$\begin{aligned} 0 \le \sum _{g \in {\mathcal {G}}} q_{g,t} - d_t ~&\perp ~ \pi _t \ge 0&\forall t \in {\mathcal {T}} \end{aligned}$$

These equations convey three important facts. (i) If a technology is used ($x_g > 0$), then the infra-marginal rents ($\sum _{t \in {\mathcal {T}}} \Delta T_t \mu _{g,t}$) earned from the short-term market prices $\pi _t$ by each technology exactly cover the investment cost $IC_g$. (ii) This means that long-term profits are zero. (iii) Furthermore, as highlighted by Boiteux, in order for the peaking units (the technology *g* with the highest $MC_g$) to recover their fixed costs, there should be at least some hours during which the system is *scarce*, meaning that the demand sets the price ($d_t < D_t$, such that $\pi _t = V_t > MC_{peak}$).

## The discrete investment problem and the lost opportunity cost

We now turn to the *discrete* version of model (1) that accounts for the lumpiness of investment. Indivisibilities in investment decisions (commissioning or decommissioning) arise naturally from the fact that power plants are large indivisible assets (e.g. nuclear or CCGT plants as well as an offshore wind park are straightforward examples). Indivisibilities also arise indirectly from economies of scale as well as learning effects. “Learning by doing” can be represented as a particular model with indivisibilities (Heuberger, Rubin, Staffell, Shah, and Mac Dowell, 2017) that appears to be of particular interest in certain policy design discussions (Newbery, 2021). The discrete investment model is as follows:

3a $$\begin{aligned} z^*_{P} = \max _{q,x,d}&\sum _{t \in {\mathcal {T}}} \Delta T_t V_t d_t - \sum _{g \in {\mathcal {G}}} \left( \sum _{t \in {\mathcal {T}}} \Delta T_t MC_g q_{g,t} + \sum _{i \in {\mathcal {I}}_g} x_{g,i} IC_{g,i} \right) \end{aligned}$$

3b $$\begin{aligned}&d_t \le \sum _{g \in {\mathcal {G}}} q_{g,t} ~~~ \forall t \in {\mathcal {T}} \end{aligned}$$

3c $$\begin{aligned}&0 \le q_{g,t} \le \sum _{i \in {\mathcal {I}}_g} P^{max}_{g,i} x_{g,i} ~~~ \forall g \in {\mathcal {G}}, ~ t \in {\mathcal {T}} \end{aligned}$$

3d $$\begin{aligned}&x_{g,i} \in \{0,1\} ~~~ \forall g \in {\mathcal {G}}, ~ i \in {\mathcal {I}}_g \end{aligned}$$

3e $$\begin{aligned}&0 \le d_t \le D_t ~~~ \forall t \in {\mathcal {T}} \end{aligned}$$

The investment decisions are modelled with the binary variables $x_{g,i}$. These stand for investment into *lumps* of capacity $P^{max}_{g,i}$ at investment cost $IC_{g,i}$, so that each market agent (or technology) *g* comes with the set of investment projects $i \in {\mathcal {I}}_g$. The real-time operations are assumed to be convex.^1^ This formulation of the discrete investment problem is similar to the one considered by Scarf (1994) or O’Neill et al. (2005). To ease notation, we shall denote the total cost of each agent for performing the production plan $(q, x)_g$ as the linear function $c_g ((q, x)_g)$ in the remainder of this paper. Thus, $c_g ((q, x)_g) = \sum _{t \in {\mathcal {T}}} \Delta T_t MC_g q_{g,t} + \sum _{i \in {\mathcal {I}}_g} x_{g,i} IC_{g,i}$. The (non-convex) production sets defined by constraints (3c)–(3d) are denoted as ${\mathcal {X}}_g$, while the (convex) consumption set defined by constraint (3e) is denoted as ${\mathcal {X}}_d$.

The short-term *marginal prices*—or *merit order* prices—are the ones stemming from the market when the investment decisions are fixed. In this paper, we are particularly interested in finding prices that support the welfare-maximizing investment.^2^ We therefore assume throughout the paper that the installed mix is the optimal investment $$x_{g,i}^{**}$$, as if a central planner were solving problem (3).

### Definition 1

(Marginal Pricing) Let $$x^{**}$$ be the values of the binary variables optimizing problem (3). The marginal prices are defined as the dual variables $$\pi ^{M}$$ obtained from solving the following (convex) problem, in which the variables *x* of problem (3) are fixed to $$x^{**}$$:

4a $$\begin{aligned} \max _{d,q}&\sum _{t \in {\mathcal {T}}} \Delta T_t V_t d_t - \sum _{g \in {\mathcal {G}}} c_g ((q, x^{**})_g) \end{aligned}$$

4b $$\begin{aligned} (\Delta T_t \pi ^{M}_t) ~&\sum _{g \in {\mathcal {G}}} q_{g,t} \ge d_t&\forall t \in {\mathcal {T}} \end{aligned}$$

4c $$\begin{aligned}&(q,x^{**})_g \in {\mathcal {X}}_g&\forall g \in {\mathcal {G}} \end{aligned}$$

4d $$\begin{aligned}&d \in {\mathcal {X}}_d \end{aligned}$$

The marginal pricing approach captures the two-stage nature of an investment cycle. The supplier first decides on the discrete decision (e.g. investing in a new power unit). The associated fixed cost is then considered as *sunk*. Thus, the price reflects the cost of operating the plant given the fixed discrete decisions (i.e. the short-term so-called *merit order*).

The concern with these marginal prices, compared to marginal pricing in the *continuous* investment problem, is that in general they do not support the optimal investment. This price alone does not support an equilibrium: some agents will have incentives to enter or to leave the market. Intuitively, the lumpiness of investment can make it socially optimal to over-dimension the investments, which in turn keeps the prices too low to render the investment profitable in the first place.^3^ This fundamental problem of the discrete investment problem was highlighted by Scarf (1994). Before illustrating it in Example 1, we proceed with some definitions that characterise the incentives of the market agents. All suppliers and consumers are assumed to be price-takers and to act so as to maximize their selfish profit.

### Definition 2

(Supplier Profit) Agent *g* is assumed to maximize its selfish profit function ${\mathcal {P}}_g$, under market price $\pi$, which is defined as follows:

5 $$\begin{aligned} \max _{(q,x)_g \in {\mathcal {X}}_g} {\mathcal {P}}_g(q,x,\pi ) \equiv \sum _{t \in {\mathcal {T}}} \pi _t \Delta T_t q_{g,t} - c_g ((q, x)_g) \end{aligned}$$

### Definition 3

(Demand Profit) The load is assumed to maximize its selfish surplus function ${\mathcal {U}}$, under market price $\pi$, defined as follows:

6 $$\begin{aligned} \max _{d \in {\mathcal {X}}_d} {\mathcal {U}}(d,\pi ) \equiv \sum _{t \in {\mathcal {T}}} \Delta T_t (V_t - \pi _t) d_t \end{aligned}$$

### Definition 4

(Competitive Walrasian Equilibrium) The allocation $(q^*, x^*, d^*)$ together with the market price $\pi$ constitute a competitive Walrasian equilibrium if

(i) for each supplier *g*, $(q^*, x^*)_g$ optimizes its profit maximization problem (5) under price $\pi$; $d^*$ optimizes the load profit maximization problem (6) under price $\pi$;
(ii) the market clears ($\sum _{g \in {\mathcal {G}}} q_{g,t}^* \ge d_t^* ~~ \forall t \in {\mathcal {T}}$).

Since the market is non-convex, a competitive equilibrium is not guaranteed to exist. Under a *centralized* production and consumption plan $(q^*, x^*)$ and $d^*$, chosen so that condition (ii) of Definition 4 is met, there may be no price that satisfies condition (i). Assuming that the private agents maximize their profit (Definitions 2 and 3), the violation of condition (i) is measured by the *long-term* lost opportunity cost.

### Definition 5

(Long-term Lost Opportunity Cost) The lost opportunity cost (*LOC*) is the difference between the selfish maximum profit if self-scheduling and the as-cleared profit (with allocation $(q^*, x^*, d^*)$) under price $\pi$. For each supplier *g*, it is expressed as:

7 $$\begin{aligned} 0 \le LOC_g(\pi ) = \overbrace{\max _{(q, x)_g \in {\mathcal {X}}_g} {\mathcal {P}}_g(q,x,\pi )}^{\text {selfish maximum profit}} - \overbrace{{\mathcal {P}}_g(q^*,x^*,\pi )}^{\text {as-cleared profit}} \end{aligned}$$

For the demand, it is expressed as:

$$\begin{aligned} 0 \le LOC_d(\pi ) = \max _{d \in {\mathcal {X}}_d} {\mathcal {U}}(d,\pi ) - {\mathcal {U}}(d^*,\pi ) \end{aligned}$$

**Fig. 1:**
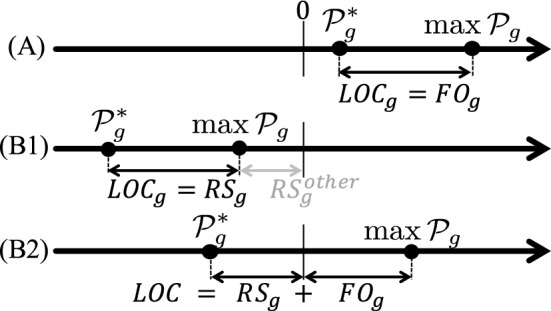
Graphical illustration of the relationship between *LOC*, *RS* and *FO*. ${\mathcal {P}}^*_g$ and $\max {\mathcal {P}}_g$ denote, respectively, the as-cleared profit and the maximum profit

This concept has been widely used in the context of pricing non-convexities in power auctions. In an investment context, the long-term lost opportunity cost measures the financial incentives that each profit-maximizing agent has to commission or decommission power plants in a way that deviates from the efficient capacity mix (the one solving problem (3)). The LOC could fruitfully be viewed as the sum of two quantities. In some cases, an LOC corresponds to a *shortfall of revenue*. For instance, a new investment that would be socially efficient, while it is unprofitable, implies that the investor would bear a shortfall of revenue. Alternatively, an installed plant that, from a social efficiency viewpoint, should stay in the market although it is unprofitable, would also face a shortfall of revenue. In a capital-intensive industry such as power production,^4^a revenue shortfall stands for a threat of not recovering investment cost. In other cases, LOC corresponds to a *foregone opportunity*. For instance, an investor who, from a social efficiency viewpoint, should restrain from investing, while his investment project is profitable, would forego an opportunity. Alternatively, if it would be socially efficient to retire an existing plant, although it is profitable, then the owner would also forego an opportunity. Mathematically, the revenue shortfall ($RS_g$) and the foregone opportunity ($FO_g$) can be related as follows to the definition of LOC (cf. Figure 1). Looking at the two terms of expression (7), there are three cases (by definition, $\max _{(q, x)_g \in {\mathcal {X}}_g} {\mathcal {P}}_g(q,x,\pi ) \ge {\mathcal {P}}_g(q^*,x^*,\pi )$):

(A) Either ${\mathcal {P}}_g(q^*,x^*,\pi ) \ge 0$, in which case there is no revenue shortfall, and the LOC is a “foregone opportunity” ($LOC_g=FO_g$), i.e. the investor does not loose money, but he could gain more by deviating from the socially efficient plan;
(B) Or ${\mathcal {P}}_g(q^*,x^*,\pi ) < 0$. In this case, there are two alternatives: (B1) Either $\max _{(q, x)_g \in {\mathcal {X}}_g} {\mathcal {P}}_g(q,x,\pi ) \le 0$, then the LOC is a revenue shortfall ($LOC_g=RS_g$); (B2) Or $\max _{(q, x)_g \in {\mathcal {X}}_g} {\mathcal {P}}_g(q,x,\pi ) > 0$. In this case, the LOC can be equivalently written as the following sum: $LOC_g (\pi ) = [\max _{(q, x)_g \in {\mathcal {X}}_g} {\mathcal {P}}_g(q,x,\pi ) - {\mathcal {P}}_g(0,0,\pi )] + [{\mathcal {P}}_g(0,0,\pi ) - {\mathcal {P}}_g(q^*,x^*,\pi )] = FO_g + RS_g$.

As highlighted in case (B2), the revenue shortfall may fruitfully be viewed as a specific “lost opportunity”, in which the as-cleared profit is negative and the “opportunity” is not to invest ($x^*=0$ and ${\mathcal {P}}_g(0,0,\pi ) = 0$).^5^ We shall reuse the notions of $RS_g$ and $FO_g$ in the sequel, especially in Sect. 6.

### Example 1

**Fig. 2:**
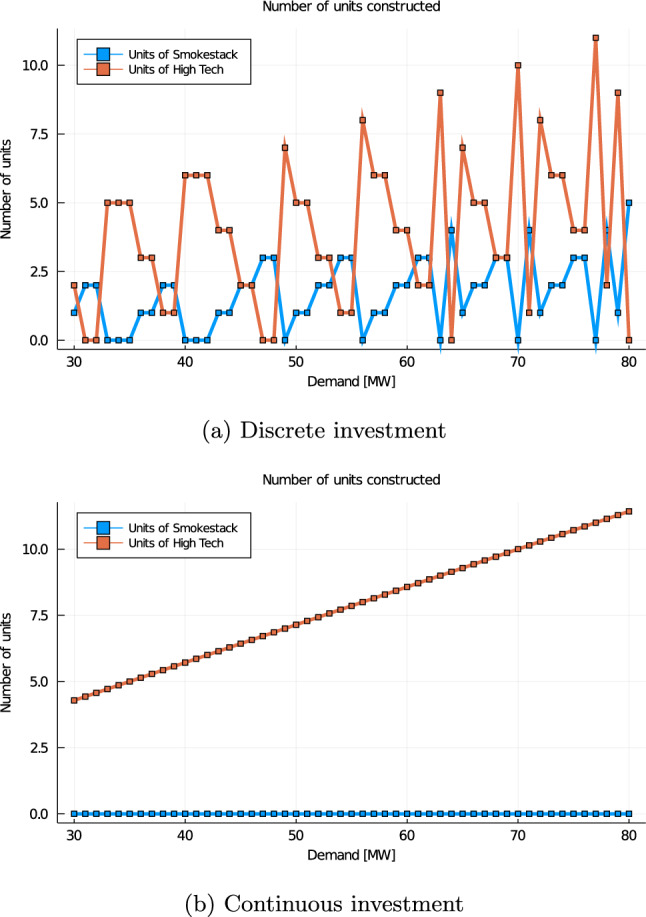
Welfare maximizing investment decisions under discrete and continuous investment as a function of the load

Consider the classic example proposed by Scarf (1994). This can be described as a discrete investment problem into two different technologies (Table 1). One technological option is Smokestack, the other is High Tech plants. A central planner solves problem (3) in order to determine the cost-minimizing number of power plants of each technology to install so as to meet the perfectly inelastic demand *D*. Figure 2a reports the cost-minimizing investment choices as a function of load. The lumpiness of investment translates into highly fluctuating investment decisions, depending on market demand. For the sake of comparison, Fig. 2b illustrates what would be the optimal expansion if the investment decisions were *continuous*. Since the average cost of the Smokestack plant is 6.3125€/MWh, while it is 6.2857€/MWh for the High Tech plant, only High Tech plants would have been built, so as to precisely meet demand (recall that the example assumes a constant uniform demand *D*). Let us now consider the case in which the demand equals to 60MWh. The optimal investment is to build 2 Smokestack plants and 4 High Tech plants. Under this allocation, the marginal price is 3€/MWh, which corresponds to the marginal cost of the Smokestack plants. The two suppliers face a lost opportunity cost (in this case, a revenue shortfall) of 106€ for the Smokestack plants and 92€ for the High Tech plants. By contrast, under continuous investment, the LOCs are zero for both technologies.

**Table 1:** Power plant data in Scarf’s example (Scarf, 1994)

	Capacity	Investment cost	Marginal cost
Technologies	[MW] ($P^{max}$)	[€/unit] (*IC*)	[€/MWh] (*MC*)
Smokestack	16	53	3
High tech	7	30	2

## The theoretical magnitude of lost opportunity costs

Indivisibilities in investment have sometimes been overlooked on the basis that, when the market size increases, “inefficiency caused by the lumpiness of generators is negligible”. As Stoft argues: “this impact of lumpiness is dramatic, but it occurs in an unrealistically small market. [...] This inefficiency declines in proportion to the size of the market.” (Stoft, 2002, pp. 130–131). In other words, the effect of indivisibilities may be dramatic in Example 1, but it tends to vanish when the size of the system increases. The same reasoning is supported by Byers and Hug (2023). In this section, we assess whether this might be theoretically true. The intuition that non-convexities would smooth out when the market size increases rests on solid theoretical foundations. A strong result was provided in the late sixties, in the theory of general equilibrium, by Arrow and Starr,^6^ in order to justify the crucial assumption of convexity that is needed for ensuring the *existence* of a competitive equilibrium. We briefly state the result of Arrow and Starr, before showing how it can be adapted to our problem statement. We then use this result in order to first derive a positive result, and then a more negative result. The settings considered by Arrow and Starr differ in two manners from our settings of Sect. 3: (i) their *pricing rule* differs from marginal pricing, and (ii) the *metric* that they use for measuring the distance from competitive equilibrium differs from LOC.

As far as the pricing scheme is concerned, in the absence of competitive prices, the question of what will be the price that prevails in the non-convex market remains open. An alternative to marginal pricing (Definition 1), consists of computing the prices from the *closest convex economy* in which a competitive equilibrium exists. Mathematically, the closest convex economy means the convex relaxation of problem (3) in which the production sets (${\mathcal {X}}_g$) are replaced by their *convex hull* ($\textrm{conv}({\mathcal {X}}_g)$).

### Definition 6

(Convex Hull Pricing) The convex hull prices $\pi ^{CH}$ are defined as the dual variables obtained from solving the following convex problem:

8a $$\begin{aligned} z_D^* = \max _{d,q,x}&\sum _{t \in {\mathcal {T}}} \Delta T_t d_t V_t - \sum _{g \in {\mathcal {G}}} c_g ((q, x)_g) \end{aligned}$$

8b $$\begin{aligned} (\Delta T_t \pi ^{CH}_t) ~&\sum _{g \in {\mathcal {G}}} q_{g,t} \ge d_t&\forall t \in {\mathcal {T}} \end{aligned}$$

8c $$\begin{aligned}&(q,x)_g \in \textrm{conv}( {\mathcal {X}}_g )&\forall g \in {\mathcal {G}} \end{aligned}$$

8d $$\begin{aligned}&d \in {\mathcal {X}}_d \end{aligned}$$

Although they do not use this nomenclature, this is the pricing approach assumed by Arrow and Starr.^7^ Let us notice that the investment costs $IC_{g,i}$ appear in problem (8) through the function $c_g ((q, x)_g)$, while they are not present in the marginal pricing problem (4), since the investment decisions are fixed.

Regarding the metric used for measuring the distance to an equilibrium, there are two options that are worth examining: either condition (ii) of Definition 4 holds—or is enforced—and (i) is violated; or condition (i) holds, in which case (ii) is violated. The first case corresponds to what has been considered in Sect. 3, in which distance to the equilibrium is measured by the LOC. The setting analysed by Arrow and Starr corresponds to the second case. It can be viewed as a purely *decentralized* setting: the producers and consumers leave or enter the market depending on the price they observe, in a manner that satisfies (i). Then, the discrepancy between demand and production—the violation of condition (ii)—is measured by the *social excess demand*.

### Definition 7

(Social Excess Demand) Let $q^\dagger _{g,t}$ and $d^\dagger _t$ be *decentralized* production and consumption plans of the private agents under price $\pi$, respecting condition (i) of Definition 4. The social excess demand (*SED*) is defined as:

9 $$\begin{aligned} SED(q^\dagger , d^\dagger ) = d^\dagger - \sum _{g \in {\mathcal {G}}} q_g^\dagger . \end{aligned}$$

### Convex hull pricing with decentralized decisions

We first consider the same setting as that assumed in the work of Arrow and Starr. Let $((q^*, x^*, d^*), \pi ^{CH})$ be the equilibrium in the *closest convex economy*, i.e. the solution of problem (8). The allocation $(q^*, x^*, d^*)$ can, in general, be infeasible. Therefore, we shall seek an allocation $(q^\dagger , x^\dagger , d^\dagger )$ that solves problems (5) and (6) under price $\pi ^{CH}$ (condition (i) in Definition 4 is met), even if it does not clear the market (condition (ii) in Definition 4 can be violated). How would this mismatch between supply and demand grow with the market size?

#### Example 2

Consider an investment problem with one single power plant technology with the following characteristics: the investment cost is 50€/MWh, the production cost is 10€/MWh and the *indivisible* size of the power plant is 100MW (i.e. investing in one plant costs 100MW$$ \times 50$$€/MWh$$ = 5,000$$€/h). Let us assume that one can invest in any non-negative integer number of power plants. We also assume that there is a single period and that the VOLL is equal to 1,000€/MWh. If the demand is $$D=250 MWh$$, the optimal allocation in the convex hull of this economy is $$x=2.5$$ and $$q=D=250$$, for which the convex hull price is $$\pi ^{CH} =60$$€/MWh. At this price, each plant is indifferent between either producing (and investing) zero, or producing at 100MWh (both production plans lead to a zero profit). There exists a decentralized decision to construct two power plants so as to produce 200MWh. On the other hand, at this price, the demand is willing to consume 250MWh. Thus, the social excess demand is 50MWh.

Intuitively, if the market grows (the demand *D* increases), the social excess demand will always be bounded by 50 MWh (which can be viewed as a measure of the non-convexity of the production set). This intuition is formally stated and proven to hold for a general case in Proposition 1, which is the translation of the Theorem of Starr and Arrow to our problem.^8^

#### Proposition 1

Let $\pi ^{CH}$ denote the convex hull prices and $(q^*, x^*,d^*)$ the associated allocation in the convex problem, where both are obtained from solving problem (8). Then, there exists an allocation $(q^\dagger , x^\dagger , d^\dagger )$ such that

(i) $(q^\dagger , x^\dagger )$ solve problem (5) under price $\pi ^{CH}$
(ii) $d^\dagger$ solves problem (6) under price $\pi ^{CH}$
(iii) The difference of social excess demand is bounded^9^$\vert SED(q^\dagger , d^\dagger ) - SED(q^*, d^*)\vert = \vert (d^\dagger - \sum _{g} q_g^\dagger ) - (d^* - \sum _{g} q_g^*) \vert \le \sqrt{\vert {\mathcal {T}} \vert } A$ with $A \ge r({\mathcal {X}}_g) ~ \forall g$.

The proof, largely inspired from the one of Arrow and Hahn (1971) that we adapt to our problem statement, is provided in Appendix 1 (which also contains all the other proofs of the paper). The Proposition shows that, under these assumptions of price and metric, the discrepancy between supply and demand, caused by the indivisibilities, is *bounded*. The bound depends upon the number of commodities that are exchanged as well as the measure of non-convexity of each production set, *but it is independent of the size of the market*. If the number of consumers and suppliers is multiplied, while keeping similar production sets, the bound remains unchanged, meaning that its ratio relative to the size of the market tends to zero.

### Convex hull pricing with centralized decisions

Let us now assume that the production and the consumption plans are decided by a central planner so that the market clears and the solution maximizes social welfare. Let $$(q^{**}, x^{**}, d^{**})$$ be the welfare-maximizing allocation, obtained from solving problem (3). The market price is again assumed to be the convex hull price $$\pi ^{CH}$$. Under this setup, condition (ii) in Definition 4 is met, while condition (i) is violated (the violation being measured by the LOC).

#### Example 3

We consider the same data as in Example 2. If $D=250$MWh, the welfare maximizing allocation is $x=3$ so that $q=D=250$ MWh. The convex hull price is $\pi ^{CH} = 60$€/MWh. At this price, the non-constructed power plants face a LOC of 0€. Two of the constructed power plants—the ones producing 100 MWh each—face a LOC of 0€. The plant at the margin, producing 50 MWh, faces a loss of 2,500€.

Intuitively, if the market grows (*D* increases), there will always be one single frustrated plant at the margin, which faces a revenue shortfall of at most 5,000€. This intuition is formally stated and proven for a general case in the following Proposition.^10^

#### Proposition 2

Let $$(q^{**},x^{**},d^{**})$$ be the welfare maximizing allocation, obtained from solving problem (3). Let $$\pi ^{CH}$$ denote the convex hull prices, obtained from solving problem (8). Then, the total lost opportunity cost is bounded:

10 $$\begin{aligned} \sum _{g \in {\mathcal {G}}} LOC_g(\pi ^{CH}) + LOC_d(\pi ^{CH}) \le \rho \vert {\mathcal {T}} \vert \end{aligned}$$

with $\rho = \max _{g \in {\mathcal {G}}} \rho _g$ and $\rho _g$ defined as follows:

11 $$\begin{aligned} \rho _g&= \max _{({\hat{q}}, {\hat{x}})_g \in \textrm{conv}({\mathcal {X}}_g)} \left\{ {\hat{c}}_g({\hat{q}}, {\hat{x}}) - c_g({\hat{q}}, {\hat{x}}) \right\} \end{aligned}$$

12 $$\begin{aligned}&{\hat{c}}_g({\hat{q}}, {\hat{x}}) = \min _{\begin{array}{c} (q,x)_g \in {\mathcal {X}}_g \\ q_{g,t} \ge {\hat{q}}_{g,t} \end{array}} c_g ((q, x)_g) \end{aligned}$$

Let us notice that, in Example 3, $\rho _g = 5,000$€. Indeed, $\vert {\mathcal {T}} \vert =1$ and a worst cost increment of 5,000€ could occur if a plant is asked to produce $\epsilon$ (the convex hull allocation is $x^*_g = \epsilon /100 \approx 0$, while a feasible allocation requires to build an entire power plant, $x_g=1$, which comes at a cost of 5,000€). Similarly to Proposition 1, the bound *does not depend on the market size*. If the market grows (increasing the load and the number of suppliers with similar production sets ${\mathcal {X}}_g$) in such a way that $z_P^* \rightarrow \infty$, since the total LOC remains bounded, its ratio with respect to the market size tends to zero, i.e. $LOC(\pi ^{CH})/z_P^* \rightarrow 0$. In other words, under convex hull prices, the lost opportunity costs do not spread over the entire market but remain contained to a small number of plants at the margin.

### Marginal pricing with centralized decisions

We now turn to the configuration considered in section 3. The production and the consumption plans are decided by a central planner but the market prices are the marginal—merit-order—prices $\pi ^{M}$, as computed from problem (4).

#### Example 4

We consider once more the same data as in Example 2. For $D=250$ MWh, the social welfare maximizing allocation is $x=3$, so that $q=D=250$ MWh. $\pi ^M=$10€/MWh. Under this price, all the plants that are not constructed are in equilibrium. But each of the three constructed plants faces a revenue shortfall of 5,000€—and not only the plant at the margin—for a total of 15,000€.

Intuitively, on this stylised example, when the demand *D* increases, the number of new plants constructed at a loss increases, and so does the lost opportunity cost which *does grow* with the size of the economy.^11^

#### Proposition 3

Let *N* be the number of times that the input of the market defined in problem (3) is duplicated, i.e. duplicating *N* times the set of suppliers $${\mathcal {G}}$$ and the load. Let $$(q^{**},x^{**},d^{**})$$ be the associated welfare-maximizing allocation and let $$\pi ^{M}$$ be the associated marginal prices. Then, in general, the lost opportunity cost is not guaranteed to be bounded, i.e. it may be that $$\lim _{N \rightarrow \infty } LOC(\pi ^{M}) = \infty $$.

Under marginal pricing, the market failure originating from indivisibilities *could* be arbitrarily large. We stress that Proposition 3 does not establish that the LOC grows to infinity *in all cases*, but simply that, in general, it is not guaranteed to be bounded, as opposed to Proposition 2. This result highlights that Propositions 1 and 2 are highly dependent on the pricing scheme that is assumed to hold in the non-convex market. Thus, under alternative prices, indivisibilities *do not* smoothen out and may have a significant impact, even in a large market. In the context of discrete investment, convex hull pricing receives a less intuitive explanation than does marginal pricing. If marginal pricing can indeed be viewed as the classic *merit order* pricing that prevails, then the LOC stemming from indivisibilities is not necessarily expected to vanish when considering a larger market size. The impact described in Proposition 3 is arguably exacerbated in Example 4 by the fact that there is a *single* peaking technology. The magnitude of the LOC under merit-order pricing will however be studied in a larger system in Sect. 6. In the meantime, we turn once again to the *two*-technology example of Scarf, Example 1, which illustrates Propositions 2 and 3, before discussing a possible solution to these lost opportunity costs in Sect. 5.

#### Example 5

**Fig. 3:**
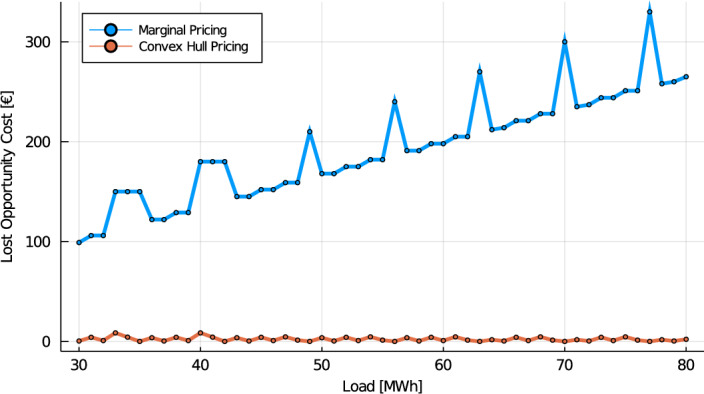
Lost opportunity costs under discrete investment, for both marginal pricing and convex hull pricing, as a function of the load

We have shown in Example 1 that the marginal price is 3€/MWh for a load of $D=60$ MWh, leading to a total lost opportunity cost of 198 €. Instead, for the same load, the convex hull price is 6.2857 €/MWh. At this price, the Smokestack and High Tech technologies face an LOC of 0.857€ and 0€ respectively. As far as the results of this section are concerned, the key observation is Fig. 3, which reports the total LOC under both pricing schemes for various load scenarios. As expected from Propositions 2 and 3, the lost opportunity cost grows with the market size under marginal pricing, while it remains bounded under convex hull pricing. The bound, computed using Proposition 2, is 53€.

## Capacity markets

What is broken by the presence of indivisibilities in the investment decisions is the possibility to achieve a perfect coordination of private agents solely by means of a uniform energy price signal (Scarf, 1994). A decentralized energy-only market does not guarantee a welfare-maximizing investment. This motivates a policy intervention for coordinating investments.

O’Neill et al. (2005) suggests viewing the issue of indivisibilities as one of market incompleteness. One commodity is *energy*, which is sold at the merit order *energy price* (indexed by time and location). Another commodity—that should also be priced—is *capacity* (the discrete investment decisions). In the approach of O’Neill, energy receives a *uniform* price, while capacity is remunerated using *discriminatory payments*. O’Neill et al. (2005) shows that there exists a set of prices $$(\pi ^{M}_t, \pi ^{C}_{i,g})$$ (remunerating energy and capacities) associated to the allocation $$(q_{g,t}^{**}, x_{g,i}^{**})$$ (solving problem (3)) that is a *competitive equilibrium*. There are two issues with this approach. Firstly, from a practical point of view, it is unclear which actual market mechanism is supposed to output these discriminatory prices. Secondly, from a theoretical point of view, the presumed price-taking behaviour of the suppliers seems to be contradicted by the mere fact that the capacity prices are discriminatory. There is essentially one single supplier for each “investment commodity”, and therefore price-taking behaviour seems like wishful thinking.^12^

In this section, we are instead interested in studying the effect of a *uniform* capacity remuneration mechanism (CRM). A capacity market is one form of long-term centralized coordination of investment decisions. The classic arguments in favour of a capacity market rest on its ability to reduce the exercise of market power and its usage as an instrument for hedging investment risk. Instead, this section investigates to what extent it could also turn out to be a means to mitigate the LOC caused by lumpy investments. As in the approach of O’Neill, the set of commodities is extended to include a remuneration for capacity. Nonetheless, the capacity auction that is considered outputs a *uniform* price.^13^ Concretely, the profit maximization problem of the market agents is now assumed to be the following:

### Definition 8

(Supplier Profit Under Energy and Capacity Prices) The agent *g* is assumed to maximize its selfish profit function ${\mathcal {P}}_g$, defined as follows:

13 $$\begin{aligned} \max _{(q,x)_g \in {\mathcal {X}}_g} {\mathcal {P}}_g(q,x,\pi ^{M},\pi ^{C}) \equiv \sum _{t \in {\mathcal {T}}} \pi ^M_t \Delta T_t q_{g,t} + \pi ^C \sum _{i \in {\mathcal {I}}_g} P^{max}_{g,i} x_{g,i} - c_g ((q, x)_g)\nonumber \\ \end{aligned}$$

The suppliers have two streams of revenue. One comes from selling energy at the marginal energy prices $\pi ^M_t$ under fixed investment. Another comes from the uniform capacity price $\pi ^C$, which remunerates their installed capacity. The capacity price comes from a capacity auction. Various designs of CRM have been considered in the literature and among practitioners, such as descending clock auctions. Both theory and experience have highlighted the advantages of sealed-bid uniform price auctions (Harbord & Pagnozzi, 2014). Our auction model can be described as follows. The suppliers submit bids that correspond to their investment costs $\sum _i x_{g,i} IC_{g,i}$, discounted by the anticipated short-term surplus from the energy market, $\sum _i P^{max}_{g,i} x_{g,i} \sum _{t \in {\mathcal {T}}_g} (\pi ^{M}_t - MC_g)$. Here, ${\mathcal {T}}_g$ are the periods for which the production of plant *g* is profitable, $\pi ^{M}_t > MC_g$. The system operator is the single buyer for the capacity target $C^{min}$, which is assumed to be inelastic.

### Definition 9

(Discrete Capacity Auction) The capacity auction minimizes the cost of satisfying the inelastic capacity demand $C^{min}$:

14a $$\begin{aligned} \min _{x} ~~&\sum _{g \in {\mathcal {G}}} \left( \sum _{i \in {\mathcal {I}}_g} x_{g,i} IC_{g,i} - \sum _{i \in {\mathcal {I}}_g} P^{max}_{g,i} x_{g,i} \sum _{t \in {\mathcal {T}}_g} \Delta T_t (\pi ^{M}_t - MC_g) \right) \end{aligned}$$

14b $$\begin{aligned} (\pi ^C) ~~&\sum _{g \in {\mathcal {G}}} \sum _{i \in {\mathcal {I}}_g} P^{max}_{g,i} x_{g,i} \ge C^{min} \end{aligned}$$

14c $$\begin{aligned}&x_{g,i} \in \{0,1\} ~~~ \forall g \in {\mathcal {G}},~ i \in {\mathcal {I}}_g \end{aligned}$$

The literature on CRMs typically focuses on continuous investment settings.^14^ This contrasts with how the CRM is implemented in certain countries, such as Belgium, where the auction accepts *only* indivisible bids. In the case of a discrete capacity auction, as in model (14), two questions arise: (i) how do we select the bids that are cleared? and (ii) how do we derive the capacity price? Harbord and Pagnozzi (2014) acknowledges these dilemmas in CRMs with indivisibilities. As far as bid selection is concerned, a natural option is to select the cost-minimizing bids, as in model (14). Proposition 4 establishes the general validity of this approach in continuous settings, while Proposition 5 indicates certain limits that are encountered under discrete settings.

### Proposition 4

Under a continuous investment model (problem (1)), with a classical “missing money” problem originating from an energy price cap, there exists a well-calibrated capacity target $$C^{min}$$ such that the optimal expansion plan $$x^{**}$$ is also a solution of the capacity auction (i.e. a continuous version of model (14)).

### Proposition 5

Under a discrete investment model (problem (3)) with long-term LOC, in some cases, the capacity cleared by the auction (i.e. solving model (14)) may differ from the optimal expansion plan $$x^{**}$$ even with a well-calibrated capacity target $$C^{min}$$.

For example, considering Scarf’s Example 1, for $D=C^{min}=60$MW, solving the auction of model (14) would lead to $x_{Smokestack}=2$ and $x_{HighTech}=4$, which corresponds to the optimal mix (cf. Figure 2a). On the other hand, solving the same auction for $D=40$MW would lead to $x_{Smokestack}=3$ and $x_{HighTech}=0$, which differs from the optimal mix. This puzzling phenomenon raises the question of how a discrete capacity market should select the bids that are cleared. In practice, alternative clearing rules have been used. For instance, according to Elia (2019), the Belgian TSO uses a “heuristic” rule to clear the CRM that even differs from cost-minimization. Moreover, system operators typically perform certain prequalification processes before solving the CRM. In Ontario and certain other systems, the system operator even solves a comprehensive capacity expansion model in order to determine the allocation of the capacity payments (Spees, Newell, and Pfeifenberger, 2013; IESO, 2023). As Proposition 5 indicates, this can be justified in certain cases.

As far as the pricing scheme is concerned, Harbord and Pagnozzi (2014) discuss various options, acknowledging the “flexibility in the definition of a market-clearing price” in a *discrete* capacity auction. They essentially focus on alternatives between the highest winning bid and the lowest losing bid. Instead, we will consider that the capacity auction relies on *convex hull pricing* (Definition 10). As highlighted in Proposition 6, this pricing scheme has the property of mitigating the long-term LOC.

### Definition 10

(Convex Hull Pricing for Capacity Auctions) The capacity price $\pi ^C$ is defined as the optimal Lagrangian multiplier.^15^ associated with the market clearing constraint in problem (14).

### Proposition 6

The uniform capacity price $\pi ^C$, as defined in Definition 10, minimizes the following lost opportunity costs:

15 $$\begin{aligned} \pi ^{C*}&= \arg \min _{\pi ^C \ge 0} \left\{ \left[ \pi ^C (\sum _{g \in {\mathcal {G}}} \sum _{i \in {\mathcal {I}}_g} P^{max}_{g,i} x_{g,i}^{**} - C^{min}) \right] \right. \nonumber \\&+ \sum _{g \in {\mathcal {G}}} \left( \max _{(q,x)_g \in {\mathcal {X}}_g } \left\{ \sum _{t \in {\mathcal {T}}} \pi ^M_t \Delta T_t q_{g,t} + \pi ^C \sum _{i \in {\mathcal {I}}_g} P^{max}_{g,i} x_{g,i} - c_g ((q, x)_g) \right\} \right. \nonumber \\&- \left. \left. \left[ \sum _{t \in {\mathcal {T}}} \pi ^M_t \Delta T_t q_{g,t}^{**} + \pi ^C \sum _{i \in {\mathcal {I}}_g} P^{max}_{g,i} x_{g,i}^{**} - c_g ((q^{**}, x^{**})_g) \right] \right) \right\} \end{aligned}$$

Here, $$(x^{**}, q^{**})$$ denotes a solution to problem (3).

A major question in the capacity auction regards the choice made by the system operator of the capacity target $$C^{min}$$. Assuming $$C^{min}=\sum _g \sum _i P^{max}_{g,i} x_{g,i}^{**}$$ (the optimum of the long term expansion problem (3)), then expression (15) corresponds to the long-term lost opportunity cost of the suppliers. More generally, as far as the first term (under bracket) in equation (15) is concerned, the following result can be established.

### Proposition 7

If $$ C^{min} \le \sum _{g \in {\mathcal {G}}} \sum _{i \in {\mathcal {I}}_g} P^{max}_{g,i} x^{**}_{g,i} $$, then the total LOC of the suppliers under both energy and capacity prices $$(\pi ^{M}, \pi ^C)$$ is lower than the LOC under the sole energy price $$\pi ^{M}$$.

Convex hull pricing in short-term auctions is known to mitigate the *short-term* LOC (Hogan & Ring, 2003). Similarly, Proposition 6 shows that CHP in a capacity auction mitigates the *long-term* LOC. However, this positive result has three limits. Firstly, although the capacity price $$\pi ^C$$ mitigates the LOC, we emphasize that it does not reduce it to zero, thus it does not entirely solve the lumpiness problem. This is, to some extent, expected. While a price cap is a distortion of the energy price that *homogeneously* affects all the suppliers, and may therefore be solved in theory by a uniform capacity price (Cramton, Ockenfels, and Stoft, 2013), investment indivisibilities distort the energy price in a manner that affects suppliers *heterogeneously*. This implies that it cannot be solved by a single instrument such as a uniform capacity price. Secondly, Proposition 6 is conditional to the fact that the bids that are cleared in the CRM are coherent with the $$x^{**}$$. As highlighted in Proposition 5, this may not always be the case. There is no straightforward solution to this problem. In Example 6, over the 50 load scenarios, the capacity mix cleared by the CRM does not equal the optimal mix in 11 scenarios (22% of the cases). This also happens in the numerical results of Sect. 6, although infrequently. Thirdly—and most importantly—, Proposition 6 is also conditional to the right calibration of the capacity targets $$C^{min}$$. For instance, as Proposition 7 emphasizes, an over-dimensioned capacity target could lead to a capacity price that exacerbates the LOC, as compared to the energy-only market, instead of mitigating it. On the other hand, a capacity target which is too low could drive the CRM price $$\pi ^C$$ to zero, thereby making the capacity auction pointless. This sensitivity of the success of a capacity auction to the calibration of the capacity target is known. De Maere d’Aertrycke et al. (2017) observe such a sensitivity in a risky environment. We consistently observe it in an environment characterised by the presence of indivisibilities. This sensitivity is revisited in the next section. To sum up, if the results of this section highlight how a CRM *may* partially resolve the incentives to invest in the context of lumpy investments, one has to be careful with the design of the capacity demand curve as well as with the capacity market clearing rule. The following example illustrates the theory that is presented in this section.

### Example 6

**Fig. 4:**
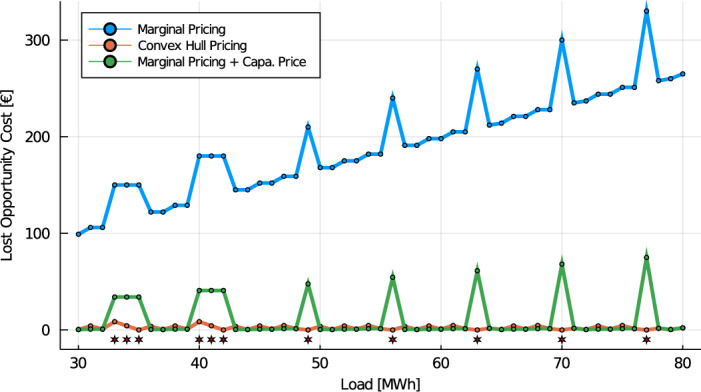
Lost opportunity costs as a function of the load, for three settlement schemes: **a** marginal pricing (Definition 1), **b** convex hull pricing (Definition 6) and **c** marginal pricing complemented with an uniform capacity price (Definition 10)

We consider once again Scarf’s Example 1. The capacity demand $$C^{min}$$ is set equal to the optimal capacity mix $$\sum _g \sum _i x_{g,i}^{**} P^{max}_{g,i}$$. So far, we have considered three settlement schemes: marginal pricing, convex hull pricing and marginal pricing complemented with a uniform capacity price. Table 2 presents the prices and LOC results for the three settlement schemes, assuming a market demand of 60 MW. Fig. 4 reports the lost opportunity costs under these three settlement schemes, for various loads. The red stars in Fig. 4 flag the load scenarios for which the bids cleared from the capacity auction differ from the optimal solution of the capacity expansion (cf. Proposition 5). In these cases, the LOC reported for the CRM assumes that the system operator intervenes for selecting the optimal bids. This could be seen as the most optimistic outcome of a uniform capacity market and is consistent with the separation of primal and dual computations in various short-term auctions, including the EU and US markets. As anticipated from Proposition 6, the addition of a capacity payment decreases the total LOC.

**Table 2:** Comparison of the pricing schemes for a demand of 60 MW

	Energy	Capacity	LOC	LOC	LOC
Settlement schemes	Price	Price	Smokestack	High tech	Total
*Marginal pricing*	3	/	106	92	198
*Convex hull pricing*	6.2857	/	0.857	0	0.857
*Marg. Price + Cap. Price*	3	3.2857	0.857	0	0.857

## Numerical simulations: the European capacity expansion problem

We now turn to the quantification of the inefficiencies resulting from the lumpiness of investment in *realistic* settings. We conduct our analysis on an investment model derived from the ENTSO-E capacity expansion model which covers the entire European system.

### The European resource adequacy assessment

ENTSO-E publishes the European Resource Adequacy Assessment (ERAA) annually. This is an analysis of the adequacy of the pan-European system which assesses European TSOs’ ability to ensure security of supply under various scenarios, for a given target year. In the 2021 ERAA study that we consider (ENTSO-E, 2021), the main target year is 2025. The ERAA has two main objectives. The first one is to assess the expected adequacy (measured with the “Loss of Load Expectation” (LOLE) [h/year]), and to compare it to the target LOLE defined by each national TSO for its country. These simulations are performed with *fixed* expected capacity, as foreseen by each national TSO. More related to the current investigation, the second objective is to undertake an Economic Viability Assessment (EVA). This is an adequacy assessment that is based on the capacity mix that results from an *economically viable* investment in power plants. Here, a capacity expansion model is solved, which includes commissioning and decommissioning decisions from the mix that is expected by the national TSOs. In our simulations, we reproduce the model of ERAA (EVA) and use its data to simulate the capacity expansion of the European system.^16^ Since the ERAA does not consider integer investment decisions, we slightly adapt the model of ERAA to turn it into a discrete investment model. With this exercise, we are particularly interested in addressing the following questions:

1. How does the introduction of lumpy investment affect the outcome of ERAA? In particular, what would be the magnitude of the LOC in such a large discrete investment model, that includes many technologies and nodes? This aims at illustrating numerically the importance of lumpiness of investment advocated in Sects. 3 and 4.
2. How would a discrete CRM affect the incentives of agents to invest in such a realistic case study? This aims at illustrating numerically the theory of Sect. 5.

We notice that ERAA also includes an analysis of the impact of a CRM. However, our analysis fundamentally differs from ERAA. Under the *continuous* setting considered by ENTSO-E, the CRM is used to solve the *missing money* problem. Indeed, the study of ENTSO-E shows that, when running the capacity expansion model (EVA) without a capacity market but with a price cap in the energy market at 15 k€/MWh, the new mix of capacities results in a slight under-investment. Concretely, the energy market alone does not lead to the “optimal” investment, as defined by the LOLE targets. In this continuous case, the capacity market is needed because of the *flawed price cap*, which is not consistent with the LOLE target.^17^. In our *discrete* case, we assume that the price cap of 15k€/MWh reflects the right VOLL, such that there is no classical “missing money”. Instead, as we work with discrete investments, the CRM plays a role of mitigating the long-term lost opportunity cost.

### The ERAA model

The detailed mathematical model of EVA is provided in the Online Appendix. In a nutshell, the EVA model includes 37 countries modelled as 59 bidding zones. The power grid is composed of HVAC and HVDC lines, although in the EVA the network constraints are represented using an ATC model. The model considers various climate years, that can be viewed as a set of 31 scenarios of load and renewable production. Not serving the load (load curtailment) is priced at *VOLL*. Production curtailment is not penalized in the objective function (the model assumes *free disposal*). The operational constraints are convex, and so are the investment decisions, which are all continuous. All the power plants of the same technology in a bidding zone are aggregated into one large virtual power plant. There are six main types of generation assets. (i) *Existing* plants can be partially retired, leading to a fixed cost reduction. (ii) *New* plants can be constructed with a fixed cost. These are the two investment decisions: continuous variables $x^{new}_g$ (commissioning) that comes with an investment cost $IC_g^{new}$ and $x^{exist}_g$ (decommissioning) that saves an investment cost $IC_g^{exist}$. (iii) Renewable assets are exogenous and therefore directly integrated in the net load. (iv) Demand response, essentially an elastic *load shedding*, is modelled as an additional convex generator at a given price. (v) Batteries are essentially a *load shifting* asset, and are modelled as a unique battery per node. (vi) There are four different types of hydro plants (all convex, described in the Online Appendix).

**Fig. 5:**
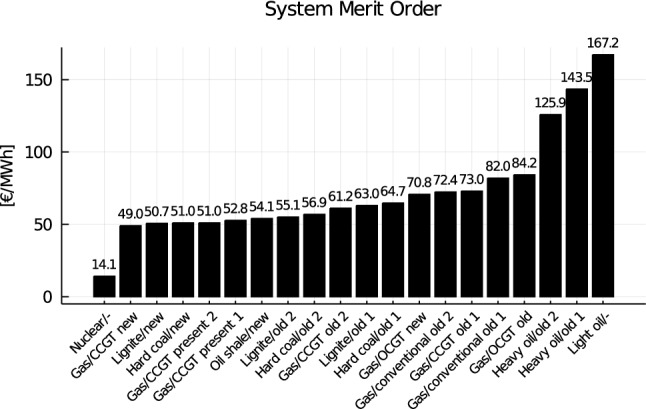
Merit order of the EVA model for the entire ENTSO-E region. Note that the model also includes a carbon tax of 40€ per ton of CO_2_ which is directly included in the operating cost of the plants

Figure 5 provides an overview of the merit order of the entire ENTSO-E system (i.e. the operational cost $MC_g$). As far as the investment decisions are concerned, each of the technologies of Fig. 5 could be decommissioned, while the commissioning decisions are limited to two technologies, CCGT and OCGT plants. To provide an order of magnitude, their investment costs are respectively 143,000 and 95,000 €/MW/y. The decommissioning of generation assets of technology *g* is limited by a parameter $RCapa^{max}_g$ that is provided by ENTSO-E. This parameter is either set to the installed capacity (meaning that the technology could be entirely decommissioned) or to a lower limit in case ENTSO-E considers it unrealistic to decommission entirely the technology (e.g. nuclear plants in France are not allowed to be decommissioned). The commissioning of new OCGT and CCGT plants is limited by a parameter $Capa^{max}_g$.

For our experiments, we have modified the ERAA model in two ways (the detailed models are in the Online Appendix):

- Since we are interested in the discrete investment model, the continuous investment decisions of ERAA are converted to integers. Concretely, investments are now the variables $x^{new}_g, x^{exist}_g \in {\mathbb {N}}$ which stand for investments in integer numbers of capacity lumps, modelled by parameters $C_g^{new}$ and $C_g^{exist}$, which are technology specific (for example, a CCGT unit is 500 MW, an OCGT unit is 300 MW... e.g. $x_{CCGT}^{new}=3$ means the entrance of 3 CCGT units of 500 MW each). The comprehensive data for parameters $C_g^{new},C_g^{exist}$ is provided in Table B2 of the Online Appendix. The energy prices $\pi ^{M}_{i,t}$ in this model are assumed to be the merit order prices of Definition 1.
- In the same spirit as Sect. 5, a capacity market is introduced. The capacity market is assumed to remunerate the capacity of flexible generation units only ($x^{new}_g$, $x^{exist}_g$), i.e. the capacity auction is limited to the thermal units (DSR, renewable or hydro plants cannot participate). As compared to Sect. 5, the capacity targets $C^{min}_i$ defined by the system operator are now indexed by the bidding zone *i*.

The model is implemented in Julia (JuMP) and is solved with Gurobi. The computations are performed on the Lemaitre3 cluster (80 nodes with two 12-core Intel SkyLake 5118 processors at 2.3 GHz and 95 GB of RAM), which is hosted at the Consortium des Equipements de Calcul Intensif (CECI).

### Numerical Results

We simulate three models: the continuous “vanilla” version of ERAA, the discrete version, and the latter complemented by capacity payments. The simulations are performed over 31 scenarios.^18^ (historical load and climate years projected to 2025 market conditions). Tables 3 and 4 report the average results of 31 scenarios as well as the detailed results for two scenarios, 2025/7 and 2025/29. The full results are in the Online Appendix. We highlight three main sets of observations relative to the comparison of discrete versus continuous investment settings, the magnitude of the long-term lost opportunity cost, and the effect of a CRM.

**Fig. 6:**
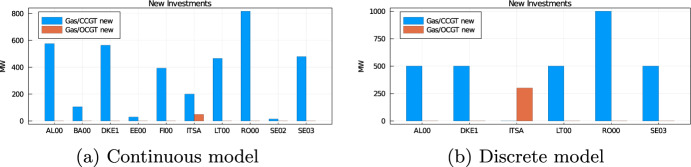
Commissioning decisions under the continuous and discrete model for scenario 2025/29

Firstly, as far as the comparison of the discrete and continuous model is concerned, Table 3 summarizes the main results from the simulations. We observe that both models can lead to fairly different results of commissioning and decommissioning decisions. Figure 6a illustrates these differences on scenario 2025/29. We observe that the commissioning of new capacities output by the continuous version of the model is reallocated across the bidding zones because of the lumpiness of the capacity. More importantly, Table 3 also reports the total cost under both continuous and discrete models. We observe that the total costs are strikingly similar. The lumpiness of investment decisions marginally affects the total system costs, which increase by a mere 0.3% on average.

**Table 3:** Comparison of the discrete and continuous results of ERAA (the full results are in the Online Appendix)

Scenarios	Total cost			Commissioning		Decommissioning		LOC
	Cont	Disc	Inc	Cont	Disc	Cont	Disc	Disc
$$\ldots $$								
2025/7	7.385e10	7.409e10	0.3%	4745	3800	37790	33000	4.91e8
$$\ldots $$								
2025/29	7.228e10	7.258e10	0.4%	3690	3300	46929	43100	4.254e8
$$\ldots $$								
Average	7.614e10	7.634e10	0.3%	7554	7048	29560	25920	1.139e9

Secondly, if the lumpiness of investment has a minor effect on *costs*, it can however significantly affect the *incentives* of the market agents. Indeed, in the continuous case, the lost opportunity cost of *all* the new and existing units is zero. This is anticipated from the theory. Under a convex model, the uniform energy prices together with the allocation of resources (investment and dispatch) form a competitive equilibrium. Instead, in the discrete case, the market agents are not in equilibrium. This is quantified in Table 3: on average, the total LOC stands for 1.5% of the total system cost. Both the new and existing power plants face incentives to deviate from the welfare maximizing allocation. We further focus on scenario 2025/29. Among all the possible comissioning (resp. decomissioning) decisions, 11% (resp. 10%) face a positive LOC. These figures show that the LOC is not contained to a few plants at the margin, but it affects the investors more broadly. At the same time, this LOC—the “burden” of investments’ indivisibilities—is not split uniformly over the entire system, but it rests on the shoulder of some private investors. For example, the revenue shortfall faced by the OCGT plant installed in ITSA (Table 5) stands for 63% of its investment cost. More generally, 67% of the effective commissioning decisions come with a revenue shortfall. On average, this revenue shortfall corresponds to 22% of the investment cost.

The lost opportunity costs are further decomposed into revenue shortfall and foregone opportunities in Table 4. A *revenue shortfall* should be read as follows. For a *new* plant, it means that it is asked to be constructed while not covering its investment cost. For an *existing* plant, it means that it is asked to not be decommissioned despite facing damages. This is further illustrated in Tables 5 and 6 which report a sample of the financial standing of various technologies per bidding zone for scenario 2025/29. In Tables 5, we observe various new plants that are commissioned (the CCGT units in zones DKE1, AL00 and RO00 as well as the OCGT in zone ITSA) while suffering losses. As far as the existing plants are concerned, in Tables 6 we observe many technologies (the table only shows a sample) that are required to stay in the market while they suffer losses (e.g. some oil plants in zone GR03 as well as CCGT units in FR00, BE00, HU00 or PT00, or some lignite plants in RS00). Similarly, twelve CCGT units in ES00 leave the market while even more units would prefer to leave the market due to the fact that they are not profitable.

A *foregone opportunity* should be understood as follows. For a *new* technology, it means that there is an incentive to invest more than what is socially optimal. Some technologies are not investing at all, while they would be profitable. Others are investing, but less than what they would given the energy price signal. For example, in Table 5, we observe that no CCGT plants in zone SE04 are commissioned while they would be profitable. In zones LT00 and SE03, one CCGT is commissioned while it is profitable and would therefore have incentives to expand. The last case means that certain new CCGT plants not only have incentives to deviate from the welfare-maximising allocation but also to earn a non-zero profit for a resource that is not scarce. They earn a “discreteness rent” of 3740 €/MW/year (for a 500MW CCGT it means 1.87 M€/year). For an *existing* plant, a foregone opportunity means that it is asked to retire while the plant is profitable. In Table 6, several CCGT plants in zone UK00 are asked to retire while they are profitable.

**Table 4:** Analysis of investor incentives decomposed into lost opportunity costs (*LOC*), revenue shortfall (*RS*) and foregone opportunity (*FO*), for the two cases including or not a capacity payment

Scenarios		Without capacity market			With capacity market		
		New units	Exist units	Total	Inelastic	Elastic	No Coord
$$\ldots $$							
	*LOC*	3.534e8	1.376e8	4.91e8	4.863e8	6.354e8	1.144e9
2025/7	*RS*	1.802e7	0.0	1.802e7	1.335e7	4.154e7	3.244e7
	*FO*	3.354e8	1.376e8	4.73e8	4.73e8	5.939e8	1.111e9
$$\ldots $$							
	*LOC*	1.052e8	3.202e8	4.254e8	5.447e7	2.678e8	1.328e9
2025/29	*RS*	6.925e7	3.171e8	3.864e8	1.312e7	4.362e7	3.012e7
	*FO*	3.599e7	3.061e6	3.905e7	4.135e7	2.242e8	1.297e9
$$\ldots $$							
	*LOC*	6.7e8	4.692e8	1.139e9	7.192e8	9.044e8	1.429e9
Average	*RS*	9.805e7	3.376e8	4.356e8	1.477e7	2.364e7	2.429e7
	*FO*	5.72e8	1.316e8	7.037e8	7.045e8	8.808e8	1.405e9

The results report three CRM settings: the inelastic capacity target, the elastic capacity demand curve and the inelastic capacity target computed without European coordination (the full results are in the Online Appendix)

These results confirm the theoretical findings from section 4: in an investment problem, the LOC resulting from indivisibilities can be significant, even in large systems. Certain market agents face incentives to invest more than what is socially optimal. In practice, they may not invest but they will then collect a positive rent for a resource that is not scarce. Other agents cannot cover both their operational and capital costs. The energy price does not play well the coordination role that it fulfils in convex settings, nor does it convey the information properly. Indeed, in the *discrete* case, some technologies end up with positive (or negative) profits. But, as Scarf emphasises, and unlike what would happen in the *continuous* case, the fact that a technology faces a positive (negative) profit does not indicate that the entire system welfare could be improved by increasing (decreasing) the investment in that technology—in fact, it would not. There is no easy solution to this issue, and as we observe later, the introduction of a capacity market can make matters worse if not properly calibrated.

**Table 5:** Detailed analysis of agents incentives for the *new* plants (commissioning) for scenario 2025/29

Zone	Technology	Investment	Profit	*LOC*	*RS*	*FO*
SE04	CCGT new	0 $\times$ 500	0.0	3.05e6	0.0	3.05e6
DKE1	CCGT new	1 $\times$ 500	$-$1.871e6	1.871e6	1.871e6	0.0
LT00	CCGT new	1 $\times$ 500	2.007e6	2.007e6	0.0	2.007e6
AL00	CCGT new	1 $\times$ 500	$-$1.626e7	1.626e7	1.626e7	0.0
FI00	CCGT new	0 $\times$ 500	0.0	1.164e7	0.0	1.164e7
EE00	CCGT new	0 $\times$ 500	0.0	7.328e6	0.0	7.328e6
RO00	CCGT new	2 $\times$ 500	$-$3.284e7	3.284e7	3.284e7	0.0
SE02	CCGT new	0 $\times$ 500	0.0	3.468e6	0.0	3.468e6
SE01	CCGT new	0 $\times$ 500	0.0	2.199e6	0.0	2.199e6
SE03	CCGT new	1 $\times$ 500	1.733e6	3.466e6	0.0	3.466e6
ITSA	OCGT new	1 $\times$ 300	$-$1.828e7	1.828e7	1.828e7	0.0
LV00	CCGT new	0 $\times$ 500	0.0	2.829e6	0.0	2.829e6

**Table 6:** Detailed analysis of agents incentives for *existing* plants (decommissioning) for scenario 2025/29 (sample)

Zone	Technology	In Place	Investment	Profit	*LOC*	*RS*	*FO*
DKE1	Light oil	529	−2 $\times$ 100	−2.871e6	2.618e6	2.618e6	0.0
GR03	Light oil	277	−0 $\times$ 100	−3.632e6	2.622e6	2.622e6	0.0
PT00	CCGT present 1	990	−0 $\times$ 450	−1.367e6	1.243e6	1.243e6	0.0
HU00	CCGT old 2	976	−0 $\times$ 400	−1.726e7	6.407e6	6.407e6	0.0
BE00	CCGT present 2	3550	−0 $\times$ 450	−6.709e6	5.953e6	5.953e6	0.0
FR00	CCGT present 2	5148	−0 $\times$ 450	−7.521e7	7.231e7	7.231e7	0.0
UK00	CCGT old 2	15010	−3 $\times$ 400	3.173e7	2.757e6	0.0	2.757e6
UK00	CCGT old 1	593	−1 $\times$ 400	146800.0	303600.0	0.0	303600.0
RS00	Lignite new	1106	−0 $\times$ 300	−2.863e7	2.33e7	2.33e7	0.0
ES00	CCGT present 1	24499	−12 $\times$ 450	−1.492e8	5.452e7	5.452e7	0.0

**Fig. 7:**
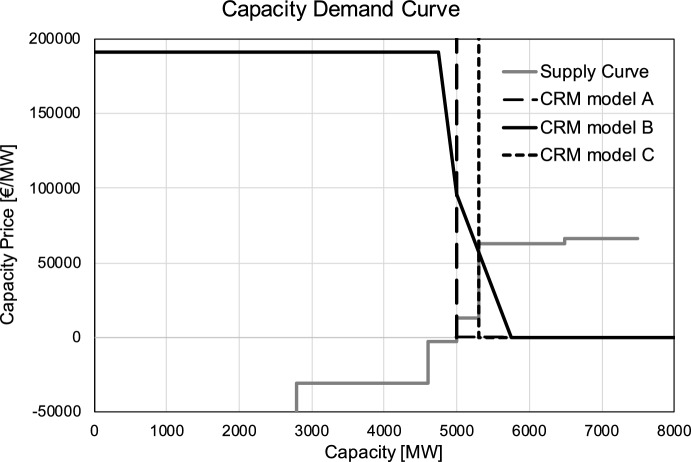
Illustration of the capacity demand curve for scenario 2025/29 in Ireland for models **A** (inelastic demand), **B** (elastic demand) and **C** (no EU-coordination). In these models, the Irish capacity price is, respectively, 0, $\sim$60,000 and $\sim$13,000 €/MW/y. As a point of comparison, the capacity price of the Belgian CRM in 2022 was $\sim$50,000 €/MW/y

The third and last aspect of our analysis regards the impact of a uniform capacity remuneration. We test three shapes of capacity demand curve:

(A) An *inelastic* capacity demand with the national capacity targets $$C^{min}_i$$ set to the *optimal* investment target with European coordination (i.e. solving the expansion problem), as in Proposition 6: $$C^{min}_i = \sum _{g \in {\mathcal {G}}_i^{new}} x^{new**}_g C_g^{new} - \sum _{g \in {\mathcal {G}}_i^{exist}} x^{exist**}_g C_g^{exist}$$, where $$x^{new**}_g$$ and $$x^{exist**}_g$$ are the optimal investment decisions derived from solving the discrete investment problem. An example is provided in Fig. 7.
(B) An *elastic* capacity demand which follows the design proposals in the literature (Cramton & Stoft, 2005; Cramton, Ockenfels, and Stoft, 2013) as well as practical applications (see the survey in Papavasiliou (2021)). An illustration is provided in Fig. 7. The demand for capacity is worth two times the entry cost of a peaker (here, an OCGT unit) up to $C^{min}_i$ (the *optimal* investment target) minus 5%. Then the valuation for capacity decreases sharply to one times the entry cost of a peaker at $C^{min}_i$, and finally becomes zero at $C^{min}_i$ plus 15%.
(C) An inelastic capacity demand, but with the $C^{min}_i$ targets computed *without* European coordination. In this case, each country computes the target capacity independently, instead of solving the European investment problem. Concretely, in order to compute $C^{min}_i$, we simulate an adapted version of the capacity expansion problem of ENTSO-E, where each country *i* is treated as an island, having to meet its national load only with domestic capacity. An illustration is provided in Fig. 7.

The right half of Table 4 presents how the lost opportunity costs are affected by the addition of a capacity market. Under CRM model (A), and as expected from Proposition 6, the capacity payments improve the overall incentives of the market agents. On average, the long-term lost opportunity costs decrease by 40% following the inclusion of a CRM, while the revenue shortfalls drop by 97%. Nonetheless, the magnitude of the impact of the CRM is heterogeneous across scenarios: for example, if the effect is significant in scenario 2025/29, it is less so in scenario 2025/7. We notice that, in our computations, the capacity price remunerates the optimal capacity mix. This could be regarded as the most optimistic result that can be achieved by a uniform price CRM. Indeed, as highlighted in Proposition 5, it may happen that the bids cleared by the CRM (as problem (14)) differ from the capacity mix optimizing problem (3). For example, in scenario 2025/29, 20 zones out of the 59 have a positive capacity price. Among these 20 zones, discrepancies between the CRM results and the optimal expansion plan occur in 15% of the cases.

The two other CRM designs stand for plausible cases of an over-dimensioned target $C^{min}_i$. They aim at evaluating the impact of a capacity price as soon as the capacity demand curve departs from the idealized settings of Proposition 6. As indicated by Proposition 7, an over-dimensioned capacity target can exacerbate the LOC. Under CRM model (B), we observe that, on average, the addition of a capacity payment still improves the incentives of market agents compared to the sole energy remuneration. In scenario 2025/29, the CRM model (A) allows to cut by ten the total lost opportunity costs. Model (B) does not perform as well, nevertheless it still lowers by 40% the total LOC compared to an energy-only settlement. However, scenario 2025/7 also highlights how model (B) can not only fail to achieve the same performance as model (A), but also perform worse than an energy-only market. In this case, the addition of a capacity payment makes the lost opportunity costs worse than they are under the sole marginal energy price. As shown in Table 4, CRM model (C) has a more disruptive effect on agents’ incentives. Since it neglects international coordination, this model tends to increase the capacity demanded in each country, thereby amplifying foregone opportunities. This highlights the benefits of having a European coordination in defining the national CRM targets, in the spirit of ERAA.

## Conclusion

In this paper, we analyse the problem of indivisibilities in investment decisions and their impact on the ability of a decentralized energy market to support efficient investments. We analyse the market failure that occurs under indivisible investment. This failure can be measured by the concept of *long-term* lost opportunity cost, which is introduced in the paper. This lost opportunity cost prevents a purely decentralized energy market to lead to a long-term equilibrium. Indivisibilities in investment have often been overlooked in the literature. A persistent argument for neglecting indivisibilities is that they supposedly vanish when the market size increases. We accurately reconstruct the underlying theoretical argument, by reviewing a classical result from the theory of general equilibrium, that we transpose to the context of power markets. We highlight that this result is only valid under specific pricing assumptions. We show that, as far as the investment problem is concerned, under the classic “merit order pricing”, the long-term lost opportunity costs can be arbitrary large. This theoretical argument is confirmed by our numerical simulations.

In order to address this market failure, we analyse the effect of introducing a CRM. We show that investment indivisibilities cast a new light on the role played by a CRM. We particularly propose the novel concept of convex hull pricing (CHP) for capacity auctions. We show that, similarly to CHP in short-term auctions, it can mitigate *long-term* lost opportunity costs. Nevertheless, we also stress the limits of a CRM: we highlight that its effect can be inconclusive—and even counter-productive—when the CRM is ill-designed. We illustrate these findings on the realistic capacity expansion model used by ENTSO-E for assessing the capacity adequacy of the European system.

As future work, we envision three possible directions. From a theoretical perspective, this work treats indivisibilities in isolation from other imperfections such as market power or risk. One theoretical inquiry is to what extent these imperfections, when combined, reinforce or mitigate each other. From a computational perspective, we have introduced indivisibilities in the capacity expansion model of ENTSO-E. This model has been work in progress for several years. A recent upgrade is the introduction of uncertainty in the model (Ávila et al., 2023). Future work could focus on combining the two features (uncertainty and indivisibility) in one model. Finally, from a policy perspective, our work focused on the interplay between investment indivisibilities and capacity markets. We have highlighted two main problems that could be explored in future works. One further development could attempt to find bounds on the capacity demand $$C^{min}$$, as safeguards against over-dimensioning. Another development could explore whether some heuristics could guide the capacity market towards the optimal mix $$x^{**}$$, without having to rely on a comprehensive capacity expansion model.

## Supplementary Information

Below is the link to the electronic supplementary material.

Supplementary file 1 (pdf 328 KB)

## Figures and Tables

**Fig. 8:**
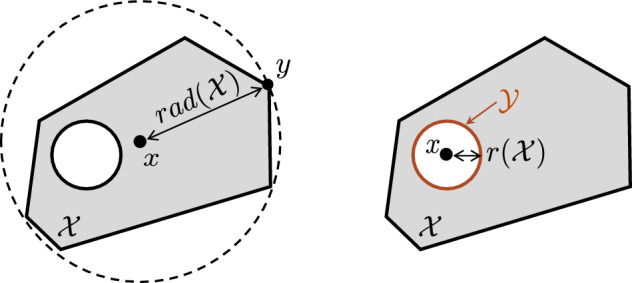
Illustration of the concepts of *radius* (left figure) and *inner radius* (right figure) of a non-convex set ${\mathcal {X}}$ (here, ${\mathcal {X}}$ is a polytope with a hole)

## Proofs of the propositions

Before establishing the proof of Proposition 1, we recall the concept of the *inner radius* of a non-convex set.

### Definition 11

Let ${\mathcal {X}} \subset {\mathbb {R}}^n$ be a compact non-convex set. The radius of this set is the radius of the smallest ball containing the set:

$$\begin{aligned} rad({\mathcal {X}}) = \min _{x \in {\mathbb {R}}^n} \max _{y \in {\mathcal {X}}} \vert x - y \vert \end{aligned}$$

For any $x \in \textrm{conv}({\mathcal {X}})$, there is a set ${\mathcal {Y}}$ spanning *x* (i.e. $x = \sum _{y \in {\mathcal {Y}}} \lambda (y) y$, with $\lambda (y) \ge 0$ and $\sum _{y \in {\mathcal {Y}}} \lambda (y) = 1$). The inner radius of ${\mathcal {X}}$ is the radius of the smallest ball including the smallest set ${\mathcal {Y}}$ spanning *x*, for any $x \in \textrm{conv}({\mathcal {X}})$:

$$\begin{aligned} r({\mathcal {X}}) = \max _{x \in \textrm{conv}({\mathcal {X}})} \min _{\begin{array}{c} {\mathcal {Y}} \subset {\mathcal {X}} \\ \text { spans } x \end{array}} rad({\mathcal {Y}}) \end{aligned}$$

Both concepts are illustrated in Fig. 8. Note that, if the production set is convex, its inner radius is clearly 0.

### Proof

(Proposition 1) Let $\pi ^{CH}$ denote the convex hull prices and $(q^*,x^*,d^*)$ the associated allocation in the relaxed problem, i.e. $(q^*,x^*)_g \in \textrm{conv}({\mathcal {X}}_g) ~ \forall g$. Since we assume that the consumption set ${\mathcal {X}}_d$ is convex, we can set $d^\dagger = d^* \in {\mathcal {X}}_d$, which indeed solves problem (6) under price $\pi ^{CH}$.

Regarding the production plan, by definition of the convex hull, $(q^*,x^*)_g = \sum _{i} \alpha _{g,i} (q^\dagger ,x^\dagger )_{g,i}$ with $\sum _i \alpha _{g,i} = 1$, $\alpha _{g,i} > 0$, for some $(q^\dagger ,x^\dagger )_{g,i} \in {\mathcal {X}}_g$. We denote ${\mathcal {Y}}_g \subset {\mathcal {X}}_g$ as the smallest set of those $(q^\dagger ,x^\dagger )_{g,i}$. Clearly, all the points $(q^\dagger ,x^\dagger )_{g,i} \in {\mathcal {Y}}_g$ solve problems (5) under the price $\pi ^{CH}$ (by optimality of the allocation $(q^*,x^*)$ in the relaxed problem).

We define $\widehat{{\mathcal {Y}}_g} = \{ q \vert (q,x) \in {\mathcal {Y}}_g \}$ (the projections of the previously defined sets ${\mathcal {Y}}_g$ over the space of variables *q*), and $z^* \in {\mathbb {R}}^{\vert {\mathcal {T}} \vert }: z_t^* = \sum _g q^*_{g,t} \in \sum _g \textrm{conv}(\widehat{{\mathcal {Y}}_g})$. By Starr’s Theorem,^19^ there exists a $z^\dagger \in \sum _g \widehat{{\mathcal {Y}}_g}$ such that $\vert z^* - z^\dagger \vert \le \sqrt{\vert {\mathcal {T}} \vert } A$. $\square$

### Proof

(Proposition 2) The proof proceeds in two steps. Firstly, from the central result of convex hull pricing theory (Gribik, Hogan, and Pope, 2007), the total lost opportunity cost corresponds to the *duality gap*:

$$\begin{aligned} \sum _{g \in {\mathcal {G}}} LOC_g(\pi ^{CH}) + LOC_d(\pi ^{CH}) = z_D^* - z_P^* \end{aligned}$$

To see this, it suffices to write the Lagrangian relaxation corresponding to $z_D^*$ and to rearrange the terms. Secondly, this duality gap is bounded.^20^ Indeed, using Minkowski’s extended formulation, problem (8) can be written as the following *linear* program:

16a $$\begin{aligned} z_D^* = \max _{d_t, \lambda _g^k}&\sum _{t \in {\mathcal {T}}} \Delta T_t d_t V_t - \sum _{g \in {\mathcal {G}}} \sum _{k \in K_g} \lambda _g^k {\hat{c}}_g^k \end{aligned}$$

16b $$\begin{aligned}&\sum _{g \in {\mathcal {G}}} \sum _{k \in K_g} \lambda _g^k {\hat{q}}_{g,t}^k \ge d_t&\forall t \in {\mathcal {T}} \end{aligned}$$

16c $$\begin{aligned}&0 \le d_t \le D_t&\forall t \in {\mathcal {T}} \end{aligned}$$

16d $$\begin{aligned}&\sum _{k \in K_g} \lambda _g^k = 1&\forall g \in {\mathcal {G}} \end{aligned}$$

16e $$\begin{aligned}&\lambda _g^k \ge 0&\forall g \in {\mathcal {G}}, ~ k \in K_g \end{aligned}$$

The set $K_g$ denotes the number of extreme points of ${\mathcal {X}}_g$ (which is assumed to be a *compact* set). Parameters ${\hat{q}}_{g,t}^k$ and ${\hat{c}}_g^k$ denote the production schedule and cost associated to each extreme point *k* of ${\mathcal {X}}_g$. There are $\vert {\mathcal {T}} \vert$ variables $d_t$ and $\vert K_g \vert$ variables $\lambda _g^k$ for each of the $\vert {\mathcal {G}} \vert$ suppliers. From constraint (16d), there is *at least* one non-zero $\lambda _g^k$ per supplier *g*. If there is *exactly one*, the solution of the relaxed problem is feasible. From the fundamental theorem of linear programming theory, there is an optimal solution that has *at least* as many constraints as variables that are tight. Therefore, we know there are *at most* $\vert {\mathcal {T}} \vert$ additional non-zero $\lambda _g^k$, meaning that at most $\vert {\mathcal {T}} \vert$ suppliers have more than one $\lambda _g^k>0$ (a production plan that is *infeasible*). Starting from the production plan that solves the convex relaxation, *at most* $\vert {\mathcal {T}} \vert$ production plans should be modified in order to obtain a *feasible* primal solution (under-approximating $z_P^*$). This modification costs *at most* $\rho _g$, which is the maximum cost resulting from turning an infeasible production plan $({\hat{q}}, {\hat{x}})_g \in \textrm{conv}({\mathcal {X}}_g)$ to a feasible production plan that supplies at least as much power. We conclude that $z_D^* - z_P^* \le \rho \vert {\mathcal {T}} \vert$. $\square$

From the proof, it is obvious that an alternative bound (tighter in case the $\rho _g$ vary significantly between the power units) is $z_D^* - z_P^* \le \sum _{g \in {\mathcal {G}}^{max}} \rho _g$, with ${\mathcal {G}}^{max}$ being the set of $\vert {\mathcal {T}} \vert$ generators with the highest $\rho _g$. Furthermore, for a convex production set ${\mathcal {X}}_g$, clearly $\rho _g = 0$.

### Proof

(Proposition 3) The Proposition follows immediately from Example 4. $\square$

The next proof is adapted from Theorem 1 in Papavasiliou (2021).

### Proof

(Proposition 4) In convex settings, the classical “missing money”, which motivates the use of a capacity market, arises because of a price cap in the energy market. Let us assume that $\pi ^{M}_t = PC$ when the system is short (load is curtailed). The continuous capacity market is:

17a $$\begin{aligned} \min _{q,x} ~~&\sum _{g \in {\mathcal {G}}} \left( x_{g} IC_{g} - \sum _{t \in {\mathcal {T}}} \Delta T_t (\pi ^{M}_t - MC_g)q_{g,t} \right) \end{aligned}$$

17b $$\begin{aligned} (\pi ^C) ~~&\sum _{g \in {\mathcal {G}}} x_{g} \ge C^{min} \end{aligned}$$

17c $$\begin{aligned}&0 \le q_{g,t} \le x_{g}&\forall g \in {\mathcal {G}}, ~ t \in {\mathcal {T}} \end{aligned}$$

17d $$\begin{aligned}&x_{g} \ge 0&\forall g \in {\mathcal {G}} \end{aligned}$$

We show that there is a well-calibrated $C^{min}$ such that the optimal solution $x^*$ of problem (1) is also a solution of auction (17). The KKT conditions of problem (17) are:

18a $$\begin{aligned} 0 \le q_{g,t} ~&\perp ~ MC_g - \pi _t^M + \mu _{g,t} \ge 0&\forall g \in {\mathcal {G}}, ~ t \in {\mathcal {T}} \end{aligned}$$

18b $$\begin{aligned} 0 \le x_g ~&\perp ~ IC_g - \pi ^C - \sum _{t \in {\mathcal {T}}} \Delta T_t \mu _{g,t} \ge 0&\forall g \in {\mathcal {G}} \end{aligned}$$

18c $$\begin{aligned} 0 \le x_g - q_{g,t} ~&\perp ~ \mu _{g,t} \ge 0&\forall g \in {\mathcal {G}}, ~ t \in {\mathcal {T}} \end{aligned}$$

18d $$\begin{aligned} 0 \le \sum _{g \in {\mathcal {G}}} x_g - C^{min} ~&\perp ~ \pi ^C \ge 0 \end{aligned}$$

Let $x^*$ be the solution of problem (1). We want to show that it satisfies (18). The energy price only differs between problems (1) (where it is called $\pi _t$) and (18) ($\pi _t^M$) during the scarcity periods ${{\mathcal {S}}}{{\mathcal {T}}}$, i.e. for $t \in {{\mathcal {S}}}{{\mathcal {T}}}$, $\pi _t^M=PC$ while $\pi _t = V_t$. We denote by $\mu _{g,t}^*$ the scarcity rents $\mu$ solving (2). Outside the scarcity periods, equations (18a) are equivalent to (2a) and $\mu _{g,t} = \mu _{g,t}^*$. During the scarcity periods $\mu _{g,t}^* = V_t - MC_g$ while equations (18a) can be written as $\mu _{g,t} = PC - MC_g = \mu _{g,t}^* - V_t + PC$. Equations (2b) can then be written equivalently as:

19 $$\begin{aligned} 0 \le x_g ~&\perp ~ IC_g - \pi ^C - \sum _{t \in {\mathcal {T}}} \Delta T_t \mu _{g,t}^* + \sum _{t \in {{\mathcal {T}}}{{\mathcal {S}}}} \Delta T_t (V_t - PC) \ge 0&\forall g \in {\mathcal {G}} \end{aligned}$$

Defining $C^{min} = \sum _{g \in {\mathcal {G}}} x_g^*$, equation (18d) implies $\pi ^C \ge 0$. Fixing $\pi ^C = \sum _{t \in {{\mathcal {T}}}{{\mathcal {S}}}} \Delta T_t (V_t - PC)$, equation (19) is then equivalent to (2b). $\square$

### Proof

(Proposition 5) The Proposition follows immediately from Example 6. $\square$

### Proof

(Proposition 6) Capacity market (14) can be written equivalently as follows:

20a $$\begin{aligned} \min _{q,x} ~~&\sum _{g \in {\mathcal {G}}} \left( \sum _{i \in {\mathcal {I}}_g} x_{g,i} IC_{g,i} - \sum _{t \in {\mathcal {T}}} \Delta T_t (\pi ^{M}_t - MC_g)q_{g,t} \right) \end{aligned}$$

20b $$\begin{aligned} (\pi ^C) ~~&\sum _{g \in {\mathcal {G}}} \sum _{i \in {\mathcal {I}}_g} P^{max}_{g,i} x_{g,i} \ge C^{min} \end{aligned}$$

20c $$\begin{aligned}&0 \le q_{g,t} \le \sum _{i \in {\mathcal {I}}_g} P^{max}_{g,i} x_{g,i}&\forall g \in {\mathcal {G}}, ~ t \in {\mathcal {T}} \end{aligned}$$

20d $$\begin{aligned}&x_{g,i} \in \{0,1\}&\forall g \in {\mathcal {G}}, ~ i \in {\mathcal {I}}_g \end{aligned}$$

The Lagrangian dual problem is then (rearranging the terms):

$$\begin{aligned}&\min _{\pi ^C \ge 0} \left\{ - C^{min} \pi ^C + \sum _{g \in {\mathcal {G}}} \max _{(q,x)_g \in {\mathcal {X}}_g} \right. \left\{ \sum _{t \in {\mathcal {T}}} \Delta T_t \pi ^{M}_t q_{g,t} + \pi ^C \sum _{i \in {\mathcal {I}}_g} P^{max}_{g,i} x_{g,i} \right. \\&\quad \left. \left. - \sum _{i \in {\mathcal {I}}_g} x_{g,i} IC_{g,i} - \sum _{t \in {\mathcal {T}}} \Delta T_t MC_g q_{g,t} \right\} \right\} \end{aligned}$$

Adding the constants $$0 = \pi ^C (\sum _{g \in {\mathcal {G}}} \sum _{i \in {\mathcal {I}}_g} P^{max}_{g,i} x_{g,i}^{**} - \sum _{g \in {\mathcal {G}}} \sum _{i \in {\mathcal {I}}_g} P^{max}_{g,i} x_{g,i}^{**})$$ and $$\sum _{g \in {\mathcal {G}}} ( - \sum _{t \in {\mathcal {T}}} \Delta T_t \pi ^{M}_t q_{g,t}^{**} + \sum _{i \in {\mathcal {I}}_g} x_{g,i}^{**} IC_{g,i} + \sum _{t \in {\mathcal {T}}} \Delta T_t MC_g q_{g,t}^{**})$$ leads to equation (15). $$\square $$

### Proof

(Proposition 7) We denote by $$LOC_g(\pi ^C, \pi ^{M})$$ the lost opportunity cost of *g* under both energy and capacity prices. $$LOC_g(0, \pi ^{M})$$ then corresponds to the LOC in the energy-only market. Denoting the optimal objective function of equation (15) by $$\xi $$, since $$\pi ^C=0$$ is a feasible solution of the optimization problem (15), we conclude that: $$\xi \le 0 \times (\sum _g \sum _i P^{max}_{g,i} x_{g,i}^{**} - C^{min}) + \sum _g LOC_g^aa{gen}(0, \pi ^{M}) = \sum _g LOC_g^{gen}(0, \pi ^{M})$$. Furthermore, if $$C^{min} \le \sum _g \sum _i P^{max}_{g,i} x^*_{g,i} $$ then $$\pi ^{C*} (\sum _g \sum _i P^{max}_{g,i} x_{g,i}^{**} - C^{min}) \ge 0$$, from which we can write $$\sum _g LOC_g^{gen}(\pi ^{C*}, \pi ^{M}) \le \xi $$. We conclude that $$\sum _g LOC_g^{gen}(\pi ^{C*}, \pi ^{M}) \le \sum _g LOC_g^{gen}(0, \pi ^{M})$$. $$\square $$

## Footnotes

1 The model neglects the non-convexities of short-term production costs, such as start-up or no-load costs.

2 In other words, the dynamics that take us from a sub-optimal mix to this optimal investment are out of scope for this paper.

3 A recent example is Finland. In April 2023 Olkiluoto-3, which is a 1600 MW nuclear unit, entered into operation and led to a significant price drop.

4 For a peaking unit or an offshore wind park, the investment cost stands for most of the total cost of the asset. For a mid-load gas-fired CCGT plant with an annualized investment cost of $\sim 80,000$€/MW/y and a production cost of $\sim 50$€/MWh with a capacity factor of $\sim$40%, the investment cost would stand for $\sim$30% of its total cost.

5 In case (B1), $\max _{(q, x)_g \in {\mathcal {X}}_g} {\mathcal {P}}_g(q,x,\pi ) <0$ may happen if possibility of inaction does not hold, i.e. if there are barriers of exit. In this case, an investor may have a shortfall of revenue, without having the possibility to act differently ($LOC_g=0$). Accounting for this subtlety requires to introduce a distinction between a “revenue shortfall that expresses a *lost opportunity*” ($RS_g$) and a “revenue shortfall that is due to a *barrier of exit*” ($RS^{other}_g$).

6 Starr (1969) shows the result for a pure exchange economy, while Arrow and Hahn (1971) show it for a more general case of an economy that includes non-convex production.

7 This happens to also be a pricing proposal that has been advocated—for other reasons—by Hogan and Ring (2003) for pricing in non-convex power auctions under the name Convex Hull (CH) Pricing. Taking the convex hull of the constraints (3c)–(3d) amounts to solving the Lagrangian relaxation of the problem in which constraint (3b) is relaxed. The convex hull prices then correspond to the associated Lagrangian multipliers. This connection with the Lagrangian dual problem justifies the label $z_D$, for denoting the *dual* objective value.

8 Note that $r(\cdot )$ denotes the *inner radius* of a set (the definition is recalled in Appendix 1). To provide an intuition to the reader, in the previous example, $r({\mathcal {X}}_g)=50$. Indeed, the set of possible investments is $\{ 0, 100\}$, meaning a ball of radius 50 MW spans any $x \in [0, 100]$.

9 Note that if $\pi _t >0~\forall t\in {\mathcal {T}}$, then we deduce that $d^* = \sum _{g} q_g^*$ and the expression becomes $\vert d^\dagger - \sum _{g} q_g^\dagger \vert \le \sqrt{\vert {\mathcal {T}} \vert } A$.

10 It recently came to the attention of the authors that a similar proposition is also provided by Chao (2019), although the proof proposed in Appendix 1 is different.

11 The reader may also want to consider these results the other way around. Let us assume that, in the same stylized example, due to some technological improvements, the plants are now available in lumps of 30 MW instead of 100 MW. The non-convex suppliers have been cut into smaller pieces so that the production sets are “less non-convex” than they used to be (as measured with the inner radius). How does the technological change affect the LOC? The reader can verify that, for D = 250 MWh, under convex hull pricing, the LOC is almost divided by three: 1,000€ (compared to the 2,500€ before the technological change). Instead, under marginal pricing, the LOC is 13,500€. This point becomes relevant as we consider distributed resources with smaller capacities in future power grids.

12 This puzzling methodological aspect connects to the well-known *Lindahl equilibrium* in public goods (Mas-Colell, Whinston, and Green, 1995). The latter also relies on the use of discriminatory prices while assuming price-taking behaviour, and has been subject to the same criticisms from economists.

13 A natural extension of the ideas developed in this section would be to consider *several* capacity targets that depends on the technology (as opposed to a *single* aggregated capacity target).

14 This is true for all the previously cited works on CRM (Fabra, 2018; Stoft, 2002; Cramton & Stoft, 2005; Cramton, Ockenfels, and Stoft, 2013; De Maere d’Aertrycke, Ehrenmann, and Smeers, 2017) that analyze a *continuous* capacity auction.

15 Considering our simple set of constraints ${\mathcal {X}}_g$, in our case, computing $\pi ^C$ is equivalent to taking the linear programming relaxation of problem (14).

16 The data can be retrieved from the website of ERAA.

17 The price cap is a key driver of the investment decisions in an energy-only market. It reflects the value at which the “lost load” is priced in the energy market (the VOLL). It should be aligned with the LOLE targets in order for the market to induce the right level of investment. Indeed, there is a strong connection between the VOLL and the LOLE (Stoft, 2002; Cramton & Stoft, 2005) Concretely, the invested capacity will be optimal if it is such that the marginal cost of an additional MW of peaking capacity (the investment cost of a peaker, $IC_{peak}$) equals the cost of one more MW of blackout ($VOLL \times LOLE$). This can be summarized by the following equation: $IC_{peak} = VOLL \times LOLE$. Note that this relationship can be derived from the KKT conditions (2) of the continuous investment problem (1), with $V_t=VOLL$. Indeed, considering the peaking units, we deduce from equation (2b) that $IC_{peak} = \sum _{t \in {\mathcal {T}}} \Delta T_t \mu _{peak,t}$. Either the peaker sets the price and $\mu _{peak,t}=0$, or demand sets the price. In the latter case, neglecting the unlikely situation in which there is a price indeterminacy, the energy price will soar up to the *VOLL*, so that $\mu _{peak,t}=VOLL-MC_{peak} \approx VOLL$. From which we conclude that $IC_g \approx VOLL \times LOLE$. See also Papavasiliou (2024).

18 This is consistent with the methodology of ENTSO-E (2021): ENTSO-E performs the simulations separately on multiple years (scenarios) and then averages the expansion plans.
