# Supplementary material for: Indivisibilities in investment and the role of a capacity market
Source: J Regul Econ. 2024 Mar 7;66(2-3):238–72. doi: 10.1007/s11149-024-09473-6 (PMC11427516; doi:10.1007/s11149-024-09473-6)
Supplement: Supplementary file 1 — (pdf 328 KB) [file 11149_2024_9473_MOESM1_ESM.pdf]

Indivisibilities in Investment and the Role of a Capacity Market — **Online Appendix**

Nicolas Stevens*, Yves Smeers and Anthony Papavasiliou

B Comprehensive ERAA mathematical model

Continuous vanilla ERAA model

In this section we present the complete model of ERAA (see also Ávila et al. (2023)). The objective is to minimize the total cost:

$$\begin{aligned}\min \sum_{g\in\mathcal{G}^{new}} x_g^{new} IC_g^{new} - \sum_{g\in\mathcal{G}^{exist}} x_g^{exist} IC_g^{exist} + \Delta T \Bigg(\sum_{\substack{i\in\mathcal{N}\\ t\in\mathcal{T}}} \xi_{i,t}^{+} VOLL \\ + \sum_{\substack{t\in\mathcal{T}\\ g\in\mathcal{G}^{new}}} MC_g^{new} q_{g,t}^{new} + \sum_{\substack{t\in\mathcal{T}\\ g\in\mathcal{G}^{exist}}} MC_g^{exist} q_{g,t}^{exist} \\ + \sum_{\substack{t\in\mathcal{T}\\ g\in\mathcal{G}^{DSR}}} MC_{g,t}^{DSR} q_{g,t}^{DSR} + \sum_{\substack{t\in\mathcal{T}, i\in\mathcal{N}\\ j\in from(i)}} |f_{i,j,t}| WC_{i,j} + \sum_{\substack{h\in\mathcal{H}\\ t\in\mathcal{T}}} s_{h,t} SP_h \Bigg)\end{aligned} \tag{1}$$

The market clearing condition is expressed as follows:

$$\begin{aligned}&\sum_{g\in\mathcal{G}_i^{new}} q_{g,t}^{new} + \sum_{g\in\mathcal{G}_i^{exist}} q_{g,t}^{exist} + \sum_{g\in\mathcal{G}_i^{DSR}} q_{g,t}^{DSR} + \sum_{g\in\mathcal{G}_i^{BAT}} (bd_{g,t} - bc_{g,t}) \\ &+ \xi_{i,t}^{+} - \xi_{i,t}^{-} + \sum_{h\in\mathcal{H}_i} q_{h,t}^{turb} - \sum_{h\in\mathcal{H}_i^{PS}} q_{h,t}^{pump} \\ &= D_{i,t} + \sum_{j\in from(i)} f_{i,j,t} - \sum_{j\in to(i)} f_{j,i,t} \quad \forall i\in\mathcal{N},\ \forall t\in\mathcal{T}\end{aligned} \tag{2}$$

*Corresponding author, e-mail: nicolas.stevens@uclouvain.be

The operational constraints of the assets are as follows:

$$0 \leq x_g^{new} \leq Capa_g^{max} \quad \forall g \in \mathcal{G}^{new} \tag{3a}$$
$$0 \leq x_g^{exist} \leq RCapa_g^{max} \quad \forall g \in \mathcal{G}^{exist} \tag{3b}$$
$$0 \leq q_{g,t}^{new} \leq x_g^{new} \quad \forall g \in \mathcal{G}^{new},\ t \in \mathcal{T} \tag{3c}$$
$$P_{g,t}^{min,exist} \leq q_{g,t}^{exist} \quad \forall g \in \mathcal{G}^{exist},\ t \in \mathcal{T} \tag{3d}$$
$$q_{g,t}^{exist} \leq P_{g,t}^{max,exist} - x_g^{exist} \frac{P_{g,t}^{max,exist}}{\max(P_{g,t}^{max,exist})} \quad \forall g \in \mathcal{G}^{exist},\ t \in \mathcal{T} \tag{3e}$$
$$0 \leq q_{g,t}^{DSR} \leq P_{g,t}^{max,DSR} \quad \forall g \in \mathcal{G}^{DSR},\ t \in \mathcal{T} \tag{3f}$$
$$F_{i,j,t}^{min} \leq f_{i,j,t} \leq F_{i,j,t}^{max} \quad \forall i \in \mathcal{N},\ j \in from(i),\ t \in \mathcal{T} \tag{3g}$$
$$0 \leq bd_{g,t}, bc_{g,t} \leq B_g^{max} \quad \forall g \in \mathcal{G}^{BAT}, t \in \mathcal{T} \tag{3h}$$
$$0 \leq bv_{g,t} \leq B_g^{capa} \quad \forall g \in \mathcal{G}^{BAT}, t \in \mathcal{T} \tag{3i}$$
$$bv_{g,t} = bv_{g,t-1} + \Delta T(B_g^{eff} bc_{g,t} - bd_{g,t}) \quad \forall g \in \mathcal{G}^{BAT},\ t \in \mathcal{T}^1 \tag{3j}$$
$$bv_{g,1} = B_g^{init} + \Delta T(B_g^{eff} bc_{g,1} - bd_{g,1}) \quad \forall g \in \mathcal{G}^{BAT} \tag{3k}$$
$$0 \leq v_{h,t}^{head} \leq V_h^{max} \quad \forall h \in \mathcal{H}^{res} \cup \mathcal{H}^{PSC} \cup \mathcal{H}^{PSO},\ t \in \mathcal{T} \tag{3l}$$
$$0 \leq q_{h,t}^{turb} \leq P_{h,t}^{max,turb} \quad \forall h \in \mathcal{H}^{res} \cup \mathcal{H}^{PSC} \cup \mathcal{H}^{PSO},\ t \in \mathcal{T} \tag{3m}$$
$$v_{h,t}^{head} = v_{h,t-1}^{head} + \Delta T(IF_{h,t} - q_{h,t}^{turb} - s_{h,t}) \quad \forall h \in \mathcal{H}^{res},\ t \in \mathcal{T}^1 \tag{3n}$$
$$v_{h,1}^{head} = V_h^{0,head} + \Delta T(IF_{h,1} - q_{h,1}^{turb} - s_{h,1}) \quad \forall h \in \mathcal{H}^{res} \tag{3o}$$
$$v_{h,t}^{head} = v_{h,t-1}^{head} + \Delta T(H_h^{eff} q_{h,t}^{pump} - q_{h,t}^{turb} - s_{h,t}) \quad \forall h \in \mathcal{H}^{PSC},\ t \in \mathcal{T}^1 \tag{3p}$$
$$v_{h,1}^{head} = V_h^{0,head} + \Delta T(H_h^{eff} q_{h,1}^{pump} - q_{h,1}^{turb} - s_{h,1}) \quad \forall h \in \mathcal{H}^{PSC} \tag{3q}$$
$$v_{h,t}^{head} = v_{h,t-1}^{head} + \Delta T(IF_{h,t} + H_h^{eff} q_{h,t}^{pump} - q_{h,t}^{turb} - s_{h,t}) \quad \forall h \in \mathcal{H}^{PSO},\ t \in \mathcal{T}^1 \tag{3r}$$
$$v_{h,1}^{head} = V_h^{0,head} + \Delta T(IF_{h,1} + H_h^{eff} q_{h,1}^{pump} - q_{h,1}^{turb} - s_{h,1}) \quad \forall h \in \mathcal{H}^{PSO} \tag{3s}$$
$$v_{h,t}^{tail} = v_{h,t-1}^{tail} + \Delta T(-H_h^{eff} q_{h,t}^{pump} + q_{h,t}^{turb}) \quad \forall h \in \mathcal{H}^{PS},\ t \in \mathcal{T}^1 \tag{3t}$$
$$v_{h,1}^{tail} = V_h^{0,tail} + \Delta T(-H_h^{eff} q_{h,1}^{pump} + q_{h,1}^{turb}) \quad \forall h \in \mathcal{H}^{PS} \tag{3u}$$
$$0 \leq q_{h,t}^{pump} \leq P_{h,t}^{max,pump} \quad \forall h \in \mathcal{H}^{PS},\ t \in \mathcal{T} \tag{3v}$$
$$\xi_{i,t}^{+}, \xi_{i,t}^{-} \geq 0 \quad \forall i \in \mathcal{N},\ t \in \mathcal{T} \tag{3w}$$

The hydro technologies are of four types:

- *Run-of-River*: a turbine without any storage, i.e. essentially a natural inflow, which is directly accounted for in the net load.
- *Reservoir*: a turbine with a reservoir, fed with inflows, that enables to choose the turbine power at each hour.
- *PS Closed*: a first pump-storage technology, composed of two reservoirs (head and tail) with pumps and turbines. The head reservoir is fed by

pumped water. The tail reservoir is fed by turbine water. There are no natural inflows.

- *PS Open*: a second pump-storage technology, which is the same as PS Closed, except that there are natural inflows feeding the head reservoir.

The model, although slightly more compact than the actual EVA model of ENTSO-E, includes *all* the features of the ENTSO-E model[1]. Table B1 provides the comprehensive nomenclature. We remark that D_t is the load net of RES production and run-of-river production. The parameter IC_g^{new} includes both annualized capital cost as well as fixed maintenance cost. The parameter IC_g^{exist} essentially includes fixed maintenance cost. The parameters MC_g^{exist} and MC_g^{new} include variable operation and maintenance cost, fuel cost as well as a CO_2 tax.

Discrete investment model

The previous model is adapted as follows. Variables x_g^{new} and x_g^{exist} are non-negative integers: $x_g^{new}, x_g^{exist} \in \mathbb{N}$. The lumps of capacity—or power plant sizes—are modelled by parameters C_g^{new} and C_g^{exist}. The data for these parameters is provided in Table B2. The sole changes with respect to the comprehensive model (3) are the constraints on investment limits (equations (3a)-(3b)), the constraints on production limits (equations (3c)-(3e)) as well as the fixed cost term in the objective (1). The investment and production constraints are now expressed as follows:

$$0 \leq C_g^{new} x_g^{new} \leq Capa_g^{max} \qquad \forall g \in \mathcal{G}^{new} \tag{4a}$$

$$0 \leq C_g^{exist} x_g^{exist} \leq RCapa_g^{max} \qquad \forall g \in \mathcal{G}^{exist} \tag{4b}$$

$$0 \leq q_{g,t}^{new} \leq C_g^{new} x_g^{new} \qquad \forall g \in \mathcal{G}^{new},\ t \in \mathcal{T} \tag{4c}$$

$$P_{g,t}^{min,exist} \leq q_{g,t}^{exist} \qquad \forall g \in \mathcal{G}^{exist},\ t \in \mathcal{T} \tag{4d}$$

$$q_{g,t}^{exist} \leq P_{g,t}^{max,exist} - \frac{x_g^{exist} C_g^{exist} P_{g,t}^{max,exist}}{\max(P_{g,t}^{max,exist})} \qquad \forall g \in \mathcal{G}^{exist},\ t \in \mathcal{T} \tag{4e}$$

The fixed costs of the objective are:

$$\sum_{g \in \mathcal{G}^{new}} x_g^{new} C_g^{new} IC_g^{new} - \sum_{g \in \mathcal{G}^{exist}} x_g^{exist} C_g^{exist} IC_g^{exist} \tag{5}$$

The energy prices $\pi_{i,t}^M$ in this model are assumed to be the merit order prices of Definition 1. The investment decisions are fixed to their optimum ($x_g^{new,**}$, $x_g^{exist,**}$) and the prices are then obtained as the dual variables of the market clearing constraints.

[1]The main "simplifications" compared to the original EVA model are the following. (i) The HVDC and HVAC lines are merged together. This is justified by the fact that the network is represented as an ATC model. (ii) Certain parameters (such as *IC* or *MC*) are pre-processed in order to make the model more compact. (iii) Finally, the data in the Turkish zone leads to outlier results of investment in Turkey. This is also acknowledged by ENTSO-E (2021). Thus, we remove Turkey from the model. This is aligned with ENTSO-E's assumption in the 2022 study.

Table B1: Nomenclature of problem (3).

**Sets**	
$\mathcal{T}$, $\mathcal{T}^1$	set of periods and $\mathcal{T}^1 = \mathcal{T} \setminus \{1\}$
$\mathcal{N}$	set of nodes
$\mathcal{G}^{new}$, $\mathcal{G}_i^{new}$	set of new plants, set of new plants in node i
$\mathcal{G}^{exist}$, $\mathcal{G}_i^{exist}$	set of existing plants, set of existing plants in node i
$\mathcal{G}^{DSR}$, $\mathcal{G}_i^{DSR}$	set of DSR units, set of DSR units in node i
$\mathcal{G}^{BAT}$, $\mathcal{G}_i^{BAT}$	set of batteries, set of batteries in node i
$\mathcal{H}^{res}$, $\mathcal{H}^{PSC}$, $\mathcal{H}^{PSO}$	set of hydro units of type Reservoir, PS Closed & PS Open
$\mathcal{H}$, $\mathcal{H}^{PS}$	set of all the hydro and $\mathcal{H}^{PS} = \mathcal{H}^{PSC} \cup \mathcal{H}^{PSO}$
**Parameters**	
ΔT	duration of a time period
D_t	net load
$Capa_g^{max}$	max capacity that can be built
$RCapa_g^{max}$	max capacity that can be retired
IC_g^{new}	investment cost of a new plant
IC_g^{exist}	investment cost of an existing plant
MC_g^{new}	operating cost of a new plant
MC_g^{exist}	operating cost of an existing plant
$MC_{g,t}^{DSR}$	price for the demand response
$P_{g,t}^{min,exist}$	min production of an existing plant
$P_{g,t}^{max,exist}$	max production of an existing plant
$P_{g,t}^{max,DSR}$	max production of a DSR plant
$F_{i,j,t}^{max}$	max flow of line from i to j
$F_{i,j,t}^{min}$	min flow of line from i to j
$WC_{i,j}$	cost of flowing power from i to j
B_g^{eff}	charging/discharging efficiency of the battery
B_g^{capa}, B_g^{max}	battery volume capacity, charging/discharging capacity
B_g^{init}	battery initial volume (in $t = 0$)
SP_h	"spill penalty" for spilling water out of the reservoir
$IF_{h,t}$	natural inflows of water (Reservoir and PS Open)
V_h^{max}	max volume of the head reservoir
$V_h^{head,0}$	initial volume of the head reservoir
$V_h^{tail,0}$	initial volume of the tail reservoir
$P_{h,t}^{max,turb}$	max power for turbine
$P_{h,t}^{max,pump}$	max power for pump (PS Open/Closed)
H_h^{eff}	efficiency of pumping (PS Open/Closed)
**Variables**	
x_g^{new}	new capacity built
x_g^{exist}	capacity being retired
$q_{g,t}^{new}$	production of a new plant
$q_{g,t}^{exist}$	production of an existing plant
$q_{g,t}^{DSR}$	production of the demand response
$f_{i,j,t}$	flow of the line from i to j
$bv_{g,t}$, $bc_{g,t}$, $bd_{g,t}$	battery volume, charge and discharge
$q_{h,t}^{turb}$, $q_{h,t}^{pump}$	turbine and pump power
$v_{h,t}^{head}$	stored volume of water in head reservoir
$v_{h,t}^{tail}$	stored volume of water in tail reservoir
$s_{h,t}$	spilled volume
$\xi_{i,t}^{+}$, $\xi_{i,t}^{-}$	load and production shedding

Table B2: Capacity lumps—or plant size—for the different technologies.

Technology	Plant Size [MW]	Technology	Plant Size [MW]
Nuclear	1000	Light oil	100
Gas/CCGT new	500	Heavy oil/old 2	300
Gas/CCGT old 2	400	Heavy oil/old 1	200
Gas/CCGT present 2	450	Oil shale/new	250
Gas/CCGT present 1	450	Hard coal/new	600
Gas/CCGT old 1	400	Hard coal/old 1	550
Gas/OCGT new	300	Hard coal/old 2	800
Gas/OCGT old	250	Lignite/new	300
Gas/conventional old 1	200	Lignite/old 1	800
Gas/conventional old 2	200	Lignite/old 2	500

Capacity market

The capacity auction model reads as follows.

$$\min_{\substack{p\geq 0\\ x\in\mathbb{N}}} \sum_{g\in\mathcal{G}^{new}} \left(x_g^{new} C_g^{new} IC_g^{new} - \Delta T \sum_{t\in\mathcal{T}} (\pi^M_{i(g),t} - MC_g^{new}) q_{g,t}^{new} \right) \tag{6a}$$

$$+ \sum_{g\in\mathcal{G}^{exist}} \left(-x_g^{exist} C_g^{exist} IC_g^{exist} - \Delta T \sum_{t\in\mathcal{T}} (\pi^M_{i(g),t} - MC_g^{exist}) q_{g,t}^{exist} \right) \tag{6b}$$

$$\sum_{g\in\mathcal{G}_i^{new}} x_g^{new} C_g^{new} - \sum_{g\in\mathcal{G}_i^{exist}} x_g^{exist} C_g^{exist} \geq C_i^{min} \tag{6c}$$

$$0 \leq x_g^{new} \leq \lfloor Capa_g^{max} / C_g^{new} \rfloor \quad \forall g \in \mathcal{G}^{new} \tag{6d}$$

$$0 \leq x_g^{exist} \leq \lfloor RCapa_g^{max} / C_g^{exist} \rfloor \quad \forall g \in \mathcal{G}^{exist} \tag{6e}$$

$$0 \leq q_{g,t}^{new} \leq C_g^{new} x_g^{new} \quad \forall g \in \mathcal{G}^{new},\ t \in \mathcal{T} \tag{6f}$$

$$P_{g,t}^{min,exist} \leq q_{g,t}^{exist} \quad \forall g \in \mathcal{G}^{exist},\ t \in \mathcal{T} \tag{6g}$$

$$q_{g,t}^{exist} \leq P_{g,t}^{max,exist} - \frac{x_g^{exist} C_g^{exist} P_{g,t}^{max,exist}}{\max(P_{g,t}^{max,exist})} \quad \forall g \in \mathcal{G}^{exist},\ t \in \mathcal{T} \tag{6h}$$

The capacity prices π_i^C are the Lagrangian multipliers associated to constraint (6c).

C Detailed numerical results

The detailed results of the summary Tables 3 and 4 are provided respectively in Tables C3 and C4. Table C3 also provides the correspondence between the scenario labels (e.g. 2025/7), used in the text of the article to denote the scenarios, and the climate years (e.g. 1989) used in the raw data of ENTSO-E.

Let's notice that among the 35 scenarios (climate years) provided by ENTSO-E, we were not computationally able to solve 4 of them (the climate years 1988, 2000, 2005, 2006) which are, therefore, not reported in the tables.

Table C3: Comparison of the discrete and continuous results of ERAA.

Climate Year	Scenario Label	Total Cost Cont.	Total Cost Disc.	Total Cost Inc.	Commissioning Cont.	Commissioning Disc.	Decommissioning Cont.	Decommissioning Disc.	LOC Disc.
1982	2025/1	7.472e10	7.493e10	0.3%	4811	5300	38233	35500	1.3032e9
1983	2025/2	7.427e10	7.453e10	0.4%	12029	11900	38020	34900	8.93e8
1984	2025/3	7.563e10	7.585e10	0.3%	4331	3500	33497	29150	1.7367e9
1985	2025/4	8.259e10	8.272e10	0.2%	23579	23900	10841	7950	1.4379e9
1986	2025/5	7.738e10	7.752e10	0.2%	10641	9600	18380	14650	8.005e8
1987	2025/6	8.214e10	8.23e10	0.2%	16320	16200	20120	16400	1.1895e9
1989	2025/7	7.385e10	7.409e10	0.3%	4745	3800	37790	33000	4.91e8
1990	2025/8	7.097e10	7.12e10	0.3%	1829	1300	35663	30750	2.5412e8
1991	2025/9	7.764e10	7.783e10	0.2%	4927	4300	23674	20550	1.207e9
1992	2025/10	7.406e10	7.427e10	0.3%	3445	3300	27520	23950	1.1151e9
1993	2025/11	7.624e10	7.644e10	0.3%	4316	3800	21012	17050	4.6549e8
1994	2025/12	7.353e10	7.38e10	0.4%	6640	5800	45503	40800	1.3519e9
1995	2025/13	7.346e10	7.372e10	0.4%	4967	4300	35929	32250	1.4473e9
1996	2025/14	7.982e10	8.001e10	0.2%	10150	9900	30945	27150	5.871e8
1997	2025/15	7.719e10	7.736e10	0.2%	6822	6800	33530	30650	3.831e9
1998	2025/16	7.472e10	7.491e10	0.3%	5600	5500	25829	23100	7.489e8
1999	2025/17	7.463e10	7.485e10	0.3%	8588	7300	32784	29000	3.2773e9
2001	2025/18	7.562e10	7.581e10	0.3%	4338	4300	29388	26700	5.246e8
2002	2025/19	7.471e10	7.491e10	0.3%	5841	5400	32895	29550	1.0566e9
2003	2025/20	8.017e10	8.04e10	0.3%	14713	13300	35522	29750	7.432e8
2004	2025/21	7.72e10	7.735e10	0.2%	7019	6300	25367	22700	5.307e8
2007	2025/22	7.214e10	7.238e10	0.3%	5689	6300	37386	34150	7.997e8
2008	2025/23	7.256e10	7.29e10	0.5%	3136	1800	46552	41300	1.4014e9
2009	2025/24	7.763e10	7.782e10	0.2%	6359	5900	18435	15750	1.383e9
2010	2025/25	8.358e10	8.376e10	0.2%	11713	10600	14991	10400	7.862e8
2011	2025/26	7.554e10	7.574e10	0.3%	6823	6800	21109	17950	2.743e9
2012	2025/27	7.811e10	7.823e10	0.2%	13708	13200	17311	14300	2.5346e8
2013	2025/28	7.75e10	7.769e10	0.2%	5919	4500	24448	20550	1.3712e9
2014	2025/29	7.228e10	7.258e10	0.4%	3690	3300	46929	43100	4.254e8
2015	2025/30	7.328e10	7.347e10	0.3%	5750	5000	33932	30350	6.574e8
2016	2025/31	7.708e10	7.725e10	0.2%	5740	5300	22735	20100	5.043e8
Average		7.614e10	7.634e10	0.3%	7554	7048	29560	25920	1.1392e9

Table C4: Analysis of the agents incentives decomposed into lost opportunity costs (LOC), revenue shortfall (RS) and foregone opportunity (FO), for the two cases including or not a capacity payment. The results report three CRM settings: the inelastic capacity target, the elastic capacity demand curve and the inelastic capacity target computed without European coordination.

Climate Year		Without capacity market			With capacity market		
		New units	Exist units	Total	Inelastic	Elastic	No Coord.
	LOC	3.396e8	9.636e8	1.303e9	1.651e8	2.603e8	9.988e8
1982	RS	1.926e8	9.6e8	1.153e9	1.468e7	2.808e7	4.155e7
	FO	1.469e8	3.521e6	1.505e8	1.505e8	2.322e8	9.573e8
	LOC	7.811e8	1.119e8	8.929e8	8.123e8	9.12e8	1.73e9
1983	RS	7.601e7	5.74e6	8.175e7	1.126e6	2.54e7	1.952e7
	FO	7.05e8	1.061e8	8.112e8	8.112e8	8.866e8	1.711e9
	LOC	6.647e8	1.072e9	1.737e9	6.055e8	8.153e8	1.87e9
1984	RS	7.247e7	1.068e9	1.14e9	8.536e6	3.135e7	3.422e7
	FO	5.923e8	4.668e6	5.969e8	5.969e8	7.839e8	1.835e9
	LOC	7.911e8	6.468e8	1.438e9	2.569e7	1.115e8	3.759e8
1985	RS	7.911e8	6.259e8	1.417e9	4.821e6	4.821e6	6.5e7
	FO	0.0	2.087e7	2.087e7	2.087e7	1.067e8	3.109e8
	LOC	4.804e8	3.201e8	8.005e8	5.978e8	7.812e8	1.089e9
1986	RS	5.245e7	1.588e8	2.113e8	0.0	0.0	1.398e6
	FO	4.28e8	1.612e8	5.892e8	5.978e8	7.812e8	1.088e9
	LOC	9.443e8	2.452e8	1.19e9	8.249e8	8.979e8	1.378e9
1987	RS	1.939e8	1.864e8	3.803e8	1.486e7	1.486e7	0.0
	FO	7.504e8	5.885e7	8.092e8	8.1e8	8.831e8	1.378e9
	LOC	3.534e8	1.376e8	4.91e8	4.863e8	6.354e8	1.144e9
1989	RS	1.802e7	0.0	1.802e7	1.335e7	4.154e7	3.244e7
	FO	3.354e8	1.376e8	4.73e8	4.73e8	5.939e8	1.111e9
	LOC	3.842e7	2.157e8	2.541e8	8.317e7	2.532e8	1.143e9
1990	RS	3.842e7	1.477e8	1.861e8	1.4e7	3.874e7	3.45e7
	FO	0.0	6.802e7	6.802e7	6.917e7	2.145e8	1.109e9
	LOC	8.765e8	3.305e8	1.207e9	1.122e9	1.301e9	1.756e9
1991	RS	2.387e7	8.05e7	1.044e8	1.975e7	2.662e7	2.662e7
	FO	8.526e8	2.5e8	1.103e9	1.103e9	1.274e9	1.73e9
	LOC	2.576e8	8.575e8	1.115e9	1.835e8	3.493e8	1.078e9
1992	RS	1.111e8	8.385e8	9.496e8	1.802e7	2.621e7	1.681e7
	FO	1.465e8	1.897e7	1.655e8	1.655e8	3.231e8	1.061e9
	LOC	3.953e8	7.019e7	4.655e8	3.965e8	5.583e8	1.386e9
1993	RS	3.947e7	4.276e7	8.223e7	1.108e7	1.044e7	1.044e7
	FO	3.558e8	2.743e7	3.833e8	3.854e8	5.479e8	1.376e9
	LOC	1.106e9	2.459e8	1.351e9	1.302e9	1.52e9	2.197e9
1994	RS	2.619e7	4.576e7	7.195e7	2.291e7	4.8e7	2.171e7
	FO	1.079e9	2.001e8	1.279e9	1.279e9	1.472e9	2.176e9
	LOC	6.289e8	8.184e8	1.447e9	6.073e8	7.575e8	1.371e9
1995	RS	6.91e7	7.831e8	8.522e8	1.219e7	4.209e7	3.381e7
	FO	5.598e8	3.527e7	5.951e8	5.951e8	7.154e8	1.337e9
	LOC	4.125e8	1.746e8	5.871e8	3.188e8	5.896e8	9.255e8

1996	*RS*	1.709e8	1.076e8	2.785e8	8.699e6	2.36e7	2.481e7
	FO	2.416e8	6.699e7	3.086e8	3.101e8	5.66e8	9.007e8
	LOC	2.769e9	1.062e9	3.83e9	3.774e9	3.779e9	4.016e9
1997	*RS*	2.477e7	6.559e7	9.036e7	3.421e7	3.421e7	2.122e7
	FO	2.744e9	9.961e8	3.74e9	3.74e9	3.745e9	3.995e9
	LOC	4.625e8	2.864e8	7.489e8	3.582e8	5.41e8	9.477e8
1998	*RS*	1.08e8	2.826e8	3.906e8	0.0	0.0	3.261e7
	FO	3.545e8	3.734e6	3.582e8	3.582e8	5.41e8	9.151e8
	LOC	2.545e9	7.323e8	3.278e9	3.267e9	3.466e9	3.697e9
1999	*RS*	1.915e7	1.555e7	3.47e7	2.379e7	2.573e7	1.554e7
	FO	2.526e9	7.168e8	3.243e9	3.243e9	3.44e9	3.681e9
	LOC	3.046e8	2.2e8	5.246e8	3.033e8	3.967e8	9.833e8
2001	*RS*	3.822e7	1.957e8	2.339e8	1.261e7	2.798e7	2.798e7
	FO	2.664e8	2.431e7	2.907e8	2.907e8	3.687e8	9.553e8
	LOC	2.839e8	7.727e8	1.057e9	4.977e8	6.292e8	1.423e9
2002	*RS*	2.321e7	5.572e8	5.804e8	2.137e7	2.276e7	2.2e7
	FO	2.607e8	2.155e8	4.762e8	4.763e8	6.065e8	1.401e9
	LOC	6.128e8	1.304e8	7.432e8	4.132e8	6.189e8	1.462e9
2003	*RS*	2.435e8	1.207e8	3.642e8	2.457e7	4.948e7	3.917e7
	FO	3.693e8	9.747e6	3.79e8	3.886e8	5.694e8	1.423e9
	LOC	3.155e8	2.152e8	5.307e8	5.307e8	6.39e8	1.086e9
2004	*RS*	1.017e7	0.0	1.017e7	1.017e7	1.017e7	1.017e7
	FO	3.053e8	2.152e8	5.205e8	5.205e8	6.288e8	1.076e9
	LOC	1.494e8	6.503e8	7.997e8	1.162e7	2.079e8	7.412e8
2007	*RS*	1.494e8	6.503e8	7.997e8	1.162e7	3.714e7	5.302e7
	FO	0.0	0.0	0.0	0.0	1.708e8	6.882e8
	LOC	1.013e9	3.884e8	1.401e9	1.319e9	1.544e9	2.431e9
2008	*RS*	0.0	1.316e8	1.316e8	4.958e7	5.062e7	1.824e7
	FO	1.013e9	2.568e8	1.27e9	1.27e9	1.494e9	2.413e9
	LOC	1.21e9	1.73e8	1.383e9	1.353e9	1.565e9	1.846e9
2009	*RS*	2.072e7	1.108e7	3.18e7	1.968e6	1.968e6	0.0
	FO	1.19e9	1.619e8	1.352e9	1.352e9	1.563e9	1.846e9
	LOC	1.196e8	6.666e8	7.862e8	1.029e8	1.238e9	6.17e8
2010	*RS*	3.189e7	6.62e8	6.939e8	1.012e7	0.0	1.012e7
	FO	8.771e7	4.592e6	9.23e7	9.274e7	1.238e9	6.068e8
	LOC	7.27e8	2.016e9	2.743e9	4.947e8	5.7e8	1.059e9
2011	*RS*	2.456e8	2.016e9	2.262e9	1.331e7	1.331e7	6.36e7
	FO	4.814e8	0.0	4.814e8	4.814e8	5.567e8	9.954e8
	LOC	1.545e8	9.896e7	2.535e8	5.663e7	1.929e8	4.525e8
2012	*RS*	1.139e8	9.277e7	2.067e8	9.862e6	9.862e6	0.0
	FO	4.058e7	6.192e6	4.677e7	4.677e7	1.83e8	4.525e8
	LOC	1.19e9	1.812e8	1.371e9	1.144e9	1.269e9	1.714e9
2013	*RS*	4.613e7	1.812e8	2.273e8	104000.0	1.576e7	1.69e7
	FO	1.144e9	0.0	1.144e9	1.144e9	1.253e9	1.697e9
	LOC	1.052e8	3.202e8	4.254e8	5.447e7	2.678e8	1.328e9
2014	*RS*	6.925e7	3.171e8	3.864e8	1.312e7	4.362e7	3.012e7
	FO	3.599e7	3.061e6	3.905e7	4.135e7	2.242e8	1.297e9
	LOC	3.969e8	2.605e8	6.574e8	5.8e8	7.717e8	1.232e9
2015	*RS*	9.872e6	1.15e8	1.248e8	4.744e7	1.837e7	1.931e7

	FO	3.871e8	1.455e8	5.326e8	5.326e8	7.533e8	1.213e9
	LOC	3.423e8	1.62e8	5.043e8	5.043e8	5.993e8	8.232e8
2016	*RS*	1.013e7	0.0	1.013e7	1.013e7	1.013e7	1.013e7
	FO	3.322e8	1.62e8	4.942e8	4.942e8	5.891e8	8.131e8
	LOC	6.7e8	4.692e8	1.139e9	7.192e8	9.044e8	1.429e9
Av.	*RS*	9.805e7	3.376e8	4.356e8	1.477e7	2.364e7	2.429e7
	FO	5.72e8	1.316e8	7.037e8	7.045e8	8.808e8	1.405e9

References

Ávila, D., A. Papavasiliou, M. Junca, and L. Exizidis. 2023. Applying high-performance computing to the european resource adequacy assessment. *IEEE Transactions on Power Systems* .

ENTSO-E 2021. European resource adequacy assessment. Technical report, https://eepublicdownloads.azureedge.net/clean-documents/sdc-documents/ERAA/ERAA_2021_Executive%20Report.pdf.
